# The greenhouse gas offset potential from seagrass restoration

**DOI:** 10.1038/s41598-020-64094-1

**Published:** 2020-04-30

**Authors:** Matthew P. J. Oreska, Karen J. McGlathery, Lillian R. Aoki, Amélie C. Berger, Peter Berg, Lindsay Mullins

**Affiliations:** 1 Department of Environmental Sciences, University of Virginia, VA, USA; 2 Department of Ecology and Evolutionary Biology, Cornell University, Ithaca, NY USA; 3 Department of Geosciences, Mississippi State University, Starkville, MS USA

**Keywords:** Ecosystem ecology, Ecosystem services, Marine biology

## Abstract

Awarding CO_2_ offset credits may incentivize seagrass restoration projects and help reverse greenhouse gas (GHG) emissions from global seagrass loss. However, no study has quantified net GHG removal from the atmosphere from a seagrass restoration project, which would require coupled C_org_ stock and GHG flux enhancement measurements, or determined whether the creditable offset benefit can finance the restoration. We measured all of the necessary GHG accounting parameters in the 7-km^2^ *Zostera marina* (eelgrass) meadow in Virginia, U.S.A., part of the largest, most cost-effective meadow restoration to date, to provide the first seagrass offset finance test-of-concept. Restoring seagrass removed 9,600 tCO_2_ from the atmosphere over 15 years but also enhanced both CH_4_ and N_2_O production, releasing 950 tCO_2_e. Despite tripling the N_2_O flux to 0.06 g m^−2^ yr^−1^ and increasing CH_4_ 8-fold to 0.8 g m^−2^ yr^−1^, the meadow now offsets 0.42 tCO_2_e ha^−1^ yr^−1^, which is roughly equivalent to the seagrass sequestration rate for GHG inventory accounting but lower than the rates for temperate and tropical forests. The financial benefit for this highly successful project, $87 K at $10 MtCO_2_e^−1^, defrays ~10% of the restoration cost. Managers should also consider seagrass co-benefits, which provide additional incentives for seagrass restoration.

## Introduction

Seagrass meadows have been identified as important sinks in the global carbon cycle, because they are highly productive systems that bury organic carbon (C_org_)^1–4^. Seagrass meadows potentially contain 4,200-8,400 Tg C_org_ in bed sediments and an additional 151 Tg C_org_ in above- and belowground biomass^5^—a significant global carbon stock threatened by accelerating seagrass habitat loss from coastal development, eutrophication, climate change, and other anthropogenic impacts^6,7^. Seagrass bed erosion following meadow collapse accelerates oxidation and remineralization of this sediment C_org_^8–10^. Global meadow loss may, therefore, release 50–330 Tg CO_2_ yr^−1^ back to the atmosphere^11^. Seagrass restoration transfers C_org_ back to the sediment^9,12,13^. However, despite increasing interest in seagrass ‘blue carbon’ and studies reporting seagrass sediment C_org_ stocks^5,13–15^, including several from restored meadows^13,16,17^, a study has yet to quantify the net greenhouse gas (GHG) removal from the atmosphere resulting from a seagrass restoration project^18^. Tokoro *et al*.^3^ provide, perhaps, the closest approximation, a net GHG removal estimate for natural seagrass meadows based on carbon flux measurements and a one-time sediment C_org_ burial rate. However, identifying the creditable GHG offset benefit requires isolating seagrass-enhanced C_org_ sequestration over time^18^, accounting for sequestered C_org_ turnover^19^, and determining whether seagrass presence also increases GHG emissions of CH_4_, N_2_O, and CO_2_ evasion associated with CaCO_3_ buried in seagrass sediment, all of which would reduce the GHG benefit from seagrass-enhanced carbon sequestration^20–22^. Seagrass GHG flux measurements, coupled with repeated measurements of C_org_ stock enhancement over time to account for C_org_ turnover within sediment and biomass carbon pools (i.e., GHG ‘stock change’), would enable calculation of seagrass-enhanced sequestration; however, there are questions about the feasibility of applying a stock change approach in a seagrass system^18^.

Prospective seagrass restoration projects currently face uncertainty about the magnitude of the GHG offset benefit they can generate, and perhaps as a result, a seagrass project has not yet applied for voluntary carbon offset-credits to help finance additional seagrass restoration^23^. Seagrass projects have been eligible to receive offset-credits since 2015, when the Verified Carbon Standard Program (the VCS, now administered by Verra) published the first seagrass offset-credit accounting framework, *VM0033: Methodology for Tidal Wetland and Seagrass Restoration*^24^. The framework has been used by countries seeking to incorporate seagrass meadows into national GHG inventories but not by individual projects. Under this methodology, the certifiable GHG offset benefit only corresponds to the net CO_2_ (or CO_2_ equivalent GHG: CO_2_e) removal from the atmosphere that can be directly attributed to a restoration project in a recognized carbon pool (i.e., negative emissions over time), minus any GHG emission increases. It is important to emphasize that this enhanced sequestration equals the CO_2_ sequestered by the restoration project (i.e., the ‘with project’ scenario) minus the background sequestration that would occur if the project did not exist (i.e., in the status quo baseline: the ‘without project’ scenario)^24,25^. The former can be measured directly; the latter must be estimated by extrapolating pre-project conditions or by comparing project and control sites over time.

**Figure 1:**
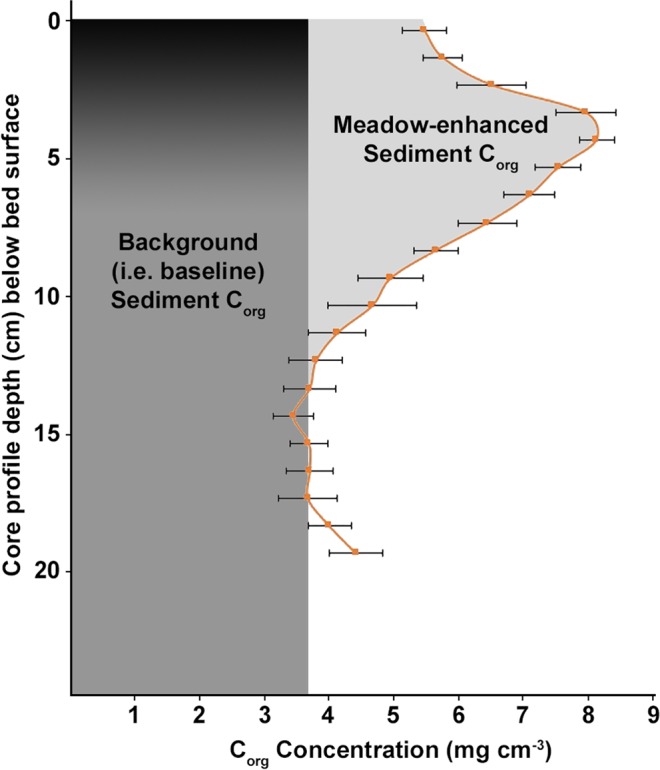
Seagrass meadow sediment C_org_ concentrations are typically highest below the surface in a region corresponding with the rhizosphere and approach the background concentration observed at unvegetated sites with increasing depth (data adapted from Greiner *et al*.^12^ and used with permission). The seagrass-enhanced sediment C_org_ stock (light gray) can be quantified by integrating the area under the profile and subtracting the background C_org_ stock that one would expect to find absent the meadow (dark gray); note that this approach does not require establishing a reference plane or quantifying bed accretion (black gradient) attributable to the meadow by sediment dating.

For seagrass restoration projects, the net GHG benefit equals CO_2_ sequestrated as enhanced sediment C_org_ (see Fig. 1: gross meadow sediment stock minus an equivalent area bare sediment stock) and the long-term average C_org_ sequestered in above- and belowground biomass within the project area, minus any enhanced GHG production^24,25^—specifically CH_4_, N_2_O, and CO_2_ evasion associated with CaCO_3_ buried in seagrass sediment^20–22,26^. Community respiration does not affect the GHG offset benefit for meadow restoration projects, because CO_2_ fixed through photosynthesis and then returned to the atmosphere through respiration is not a net flux of CO_2_ to the atmosphere. Enhanced respiration could, however, adversely affect a seagrass conservation project attempting to avoid the remineralization of sequestered C_org_ stocks. As noted above, the offset benefit from seagrass biomass sequestration over interannual timescales corresponds to the average, annual standing biomass stock, not peak biomass. This average reflects loss and turnover due to herbivory, senescence, export, and, in some cases, harvest or other disturbances. Some of the exported seagrass carbon may remain sequestered at deep ocean depositional sites^27^, and some is deposited along the coastline as wrack on beaches, marshes, and on tidal flats. The VCS and other offset crediting standards conservatively assume that exported biomass is decomposed and returns to the atmosphere as CO_2_.

The offset-credit methodology recommends measuring the sediment C_org_ stock repeatedly over time to quantify sequestered C_org_ enhancement (i.e., stock change), rather than measuring the C_org_ stock to an arbitrary depth on a single occasion or estimating C_org_ accumulation from burial rates^25^. This is because seagrass sediment C_org_ stock estimates^15,28,29^ and burial rates^2,30,31^ likely overestimate net CO_2_ removal from the atmosphere due to uncertainties with dating techniques for sediment accretion over relatively short time scales (decades)^18^. These estimates also include allochthonous carbon (C_org_ fixed outside the project area) that is excluded from GHG offset accounting methodologies and background C_org_ that would be sequestered in the area in the baseline scenario (see Supplement)^18,32^. This study shows how repeated stock change measurements can provide a more reliable approach for assessing how meadow presence enhances sediment C_org_ accumulation and how remineralization, especially in the upper mixed layer of the sediment^18,19,33^, affects this C_org_ to determine sequestration for offset-credit accounting^34^.

Uncertainty about how seagrass restoration affects CH_4_ and N_2_O fluxes represents a data gap for prospective restoration projects. The VCS defines the *de minimis* threshold at <5% of the GHG benefit; fluxes of CH_4_ and N_2_O lower than this are discounted in offset accounting^24^. Given their higher global warming potentials relative to CO_2_, a marginal increase in either CH_4_ or N_2_O production could substantially reduce the net GHG benefit from meadow restoration^35–37^. Emissions of CH_4_ and N_2_O from seagrass systems were earlier assumed to be negligible^38,39^, because H_2_S produced by sulfate reduction oxidizes CH_4_ in marine sediments^40,41^ and seagrass nitrogen demand limits N_2_O efflux^42^. Oremland^43^ and Moriarty *et al*.^44^ reported very low seagrass methane fluxes, and studies have documented high sulfate reduction in seagrass beds^44–46^. However, several recent studies have determined that CH_4_ and N_2_O enhancement partially offsets the ‘blue carbon’ benefit in mangrove and marsh systems^37,47–49^. A recent review found that CH_4_ fluxes in seagrass systems varied considerably, from 1.25–401.50 μmol CH_4_ m^−2^ d^−1^, and were lower on average than mangrove and salt marsh habitats^48^ (Table 1). One study has suggested that seagrass sediments may limit N_2_O release (Table 2), but the only available N_2_O data from a seagrass system derives from sediment core incubations^50^.

**Table 1:** Reported CH_4_ flux data for seagrass systems.

Location	Seagrass	Method	CH_4_ Flux (µmol m^−2^ hr^−1^)	Notes	Reference
Florida Keys, FL, USA	*Thalassia testudinum*	Benthic chambers and incubations	1.81–1.86		^43^
Bimini, Bahama Island	*Syringodium* sp.	Benthic chambers and incubations	0.14–0.47		^43^
Moreton Bay, Australia	*Zostera capricorni*	*In vitro* incubations	14.5	Est. for top 20 cm of bed	^44^
Red Sea	Multispecies: *Thalassodendron ciliatum*, *Cymodocea serrulata*, *Halodule uninervis*, etc.	Core incubations	0.004–23.6	Salinity range = 37.98–42.29	^49^
Ria Formosa Lagoon, Portugal	*Zostera noltii*	Benthic chambers	4.4	Aerial exposure at night	^82^
Ria Formosa Lagoon, Portugal	*Zostera noltii*	Benthic chambers	6.9	Aerial exposure during day	^82^
Ria Formosa Lagoon, Portugal	*Zostera noltii*	Benthic chambers	9.0–30	During tidal flooding	^82^
Ria Formosa Lagoon, Portugal	*Zostera noltii*	Benthic chambers	4.4–71(mean = 12.8)		^82^
Florida Bay, FL, USA	*Thalassia testudinum*	Benthic chambers and porewater samples	0.567	Dead seagrass areas in winter	^83^
Florida Bay, FL, USA	*Thalassia testudinum*	Benthic chambers and porewater samples	14.21	Live seagrass areas in fall	^83^
Cape Lookout Bight, NC, USA	*Zostera marina and Halodule sp*.	Core extraction, centrifuging, porewater sampling	20–2000	Seagrass not specifically studied but occurs in the general study area	^84^
Arcachon Bay, France	*Zostera noltii*	Benthic chambers	1.6–32.7	Sed-water flux with seasonal variation	^85^
Chilika Lagoon, India	Multispecies: *Halodule* spp., *Halophila* spp.	Open water and sediment samples	4.17, 5.6	Wet and dry season averages	^86^
Tomales Bay, CA, USA	(*Zostera marina*)	Benthic chambers	2.08	Summer eelgrass bed	^87^
Tomales Bay, CA, USA	(*Zostera marina*)	Benthic chambers	0.896	Winter eelgrass bed	^87^
**South Bay, VA, USA**	***Zostera marina***	**Benthic chambers**	**13.110** ± **4.570**	**Seagrass spring average**	**This study**
**South Bay, VA, USA**	***Zostera marina***	**Benthic chambers**	**3.136** ± **1.307**	**Seagrass summer average**	**This study**
**South Bay, VA, USA**	***Zostera marina***	**Benthic chambers**	**0.845** ± **0.255**	**Seagrass fall average**	**This study**
**South Bay, VA, USA**	***Zostera marina***	**Benthic chambers**	**5.697**	**Seagrass 9-month average**	**This study**
**South Bay, VA, USA**	***Zostera marina***	**Benthic chambers**	**1.778** ± **0.930**	**Bare spring average**	**This study**
**South Bay, VA, USA**	***Zostera marina***	**Benthic chambers**	**0.050** ± **0.021**	**Bare summer average**	**This study**
South Bay, VA, USA	***Zostera marina***	**Benthic chambers**	**0.387** ± **0.104**	**Bare fall average**	**This study**
South Bay, VA, USA	***Zostera marina***	**Benthic chambers**	**0.739**	**Bare 9-month average**	**This study**

**Table 2:** Reported N_2_O flux data for seagrass systems.

Location	Seagrass	Method	N_2_O Flux (µmol m^−2^ hr^−1^)	Notes	Reference
Nanwan Bay, Taiwan	*Thalassia hemprichii*, *Halodule uninervis*	Sediment incubations	0.3–2.2*	12-hr incubations	^42^
Lake Akkeshi, Japan	*Zostera marina*	Sediment incubations	(0.009–0.022 µmol L^−1^)	Concentrations following 7-day incubations	^50^
South Bay, Virginia, USA	***Zostera marina***	**Benthic chambers**	**0.378** ± **0.184**	**Seagrass spring average**	**This study**
South Bay, Virginia, USA	***Zostera marina***	**Benthic chambers**	**0.043** ± **0.013**	**Seagrass summer average**	**This study**
South Bay, Virginia, USA	***Zostera marina***	**Benthic chambers**	**0.039** ± **0.007**	**Seagrass fall average**	**This study**
South Bay, Virginia, USA	***Zostera marina***	**Benthic chambers**	**0.153**	**Seagrass 9-month average**	**This study**
South Bay, Virginia, USA	***Zostera marina***	**Benthic chambers**	**0.120** ± **0.073**	**Bare spring average**	**This study**
South Bay, Virginia, USA	***Zostera marina***	**Benthic chambers**	**0.003** ± **0.002**	**Bare summer average**	**This study**
South Bay, Virginia, USA	***Zostera marina***	**Benthic chambers**	**0.046** ± **0.013**	**Bare fall average**	**This study**
South Bay, Virginia, USA	***Zostera marina***	**Benthic chambers**	**0.057**	**Bare 9-month average**	**This study**

^*^µmol g wet wt^−1^ hr^−1^.

Without adequate data to quantify the net GHG benefit from seagrass restoration, the VCS allows projects to use the emission factor for seagrass established by the Intergovernmental Panel on Climate Change (IPCC) for national GHG inventory accounting, 0.43 t C ha^−1^ yr^−1^ ^51^, even in areas where regional or local estimates for some parameters are available^24,34^. This default factor may over/underestimate the net GHG benefit. The number derives from only two studies of *Posidonia oceanica*, a seagrass species that generates unusually high sediment C_org_ stocks, and does not account for the baseline sediment C_org_ stock, allochthonous carbon, or the enhancement of GHG fluxes^52,53^.

**Figure 2:**
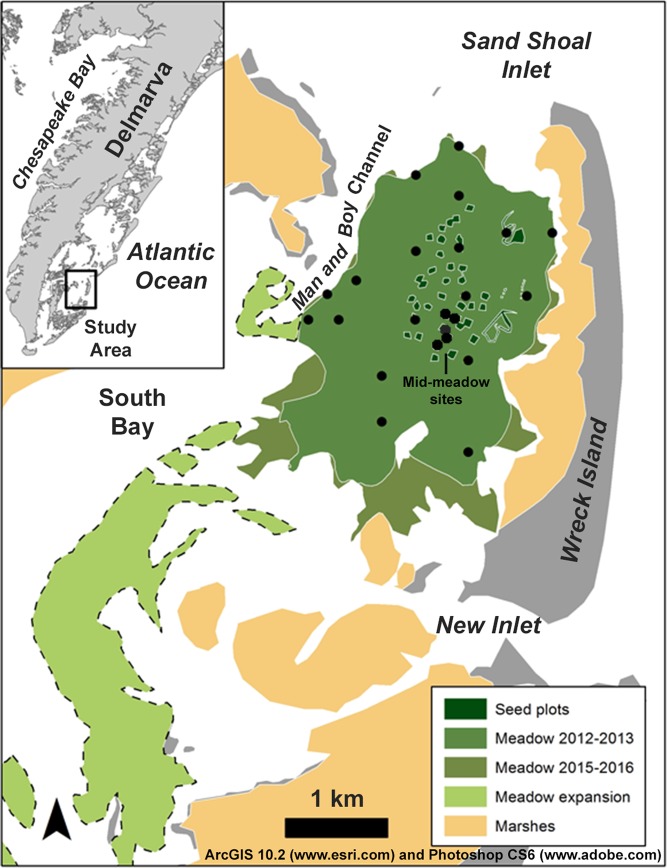
The South Bay, Virginia, study area, showing the locations of biomass and sediment C_org_ sample sites (black circles), original restoration seed plots (established in 2000–2001: Orth *et al*.^70^, the central meadow extent prior to sampling in 2013, and the expanded meadow extent prior to sampling in 2016. Meadow expansion areas to the west and south (light green areas enclosed by dashed lines) were excluded from the net GHG benefit calculations in this study. The figure was created in ArcGIS 10.2 (www.esri.com) and Photoshop CS6 (www.adobe.com).

This study is the first study to calculate net GHG removal by a seagrass restoration project based on measured data for all of the parameters required by the VCS accounting framework^23,24^, making this the first verification that seagrass systems provide a creditable GHG offset benefit. We leveraged the long-term seagrass restoration and monitoring effort in the Virginia, U.S.A., coastal bays, which is acknowledged as the world’s largest successful seagrass restoration to date. Our study focused on the 7 km^2^ *Zostera marina* (eelgrass) meadow in South Bay (Fig. 2). We undertook this work to address two urgent GHG accounting questions: 1) does seagrass restoration increase GHG fluxes that adversely impact the net GHG benefit, and 2) is the IPCC seagrass restoration default factor conservative for GHG accounting^51^? No other study has attempted to apply these comprehensive GHG accounting methods to a seagrass system before. This study, therefore, establishes a benchmark for expectations about seagrass ‘blue carbon’ finance potential, because the South Bay meadow likely remains the least expensive meadow restoration on a cost per area basis^17,54^. It represents a best-case scenario for potentially financing restoration through offset-crediting.

## Results

### Enhanced carbon sequestration

**Figure 3:**
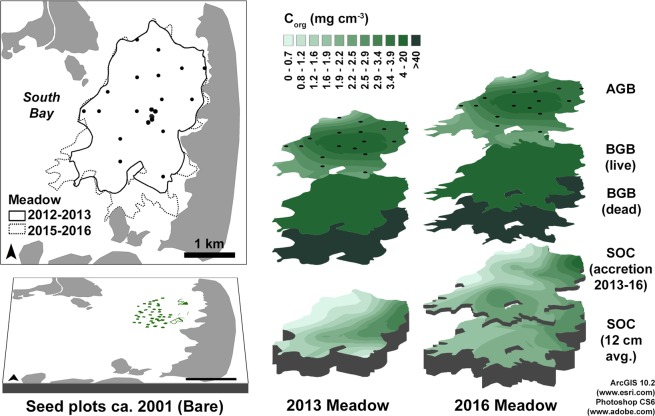
Sequestered GHG pools (aboveground biomass - AGB, belowground biomass - BGB, and net sediment C_org_ – SOC) in 2013 and in 2016 resulting from seagrass restoration; maps generated by kriging data measured at sample sites (n = 21: circles in inset map); note that the bed volume has changed over time due to both meadow expansion and accretion (see Supplement). The mid-meadow SOC decline in the 2016 accreted interval reflects a local seagrass die-off event in 2015. The figure was created in ArcGIS 10.2 (www.esri.com) and Photoshop CS6 (www.adobe.com).

With repeated stock change measurements, we observed significant C_org_ stock enhancement at the meadow scale resulting from increasing C_org_ concentrations within the bed, seagrass-enhanced bed accretion, and meadow expansion. The meadow-wide, net sediment C_org_ sequestration attributable to the restoration increased from 1,130 t C_org_ in 2013 to 2,010 t in 2016 (Table 3; Fig. 3). Note that these values are stocks relative to a known baseline that represents the ‘without restoration project’ scenario, not rates, which can be obtained by dividing the stock by a time interval. Approximately 280 t of this 880 t C_org_ stock increase occurred in the top 2 cm of the bed, which was likely deposited between 2013 and 2016 (see Supplement discussion of accretion); the remaining 600 t accumulated within the bed between 2013 and 2016. The 2013 meadow stored an average of 196 g C_org_ m^−2^ and the 2016 meadow stored an average of 292 g C_org_ m^−2^. The 2013 enhanced stock took 12 years to accumulate. Between 2013 and 2016, the enhanced sediment C_org_ stock almost doubled, indicating that the sequestration rate also increased. Meadow C_org_ sequestration in sediments was 346 t CO_2_ yr^−1^ from 2001–2013 and 1070 t CO_2_ yr^−1^ from 2013–2016.

**Table 3:** Sequestered CO_2_ stocks (negative values), cumulative GHG emissions, and the net GHG benefit from the South Bay meadow in 2013 and in 2016; all values are Mt CO_2_ equivalent units. Gross values = observed seagrass meadow-scale stocks; net values = seagrass meadow stock enhancement above the baseline (gross seagrass stocks – equivalent area bare stocks; aboveground biomass - AGB, belowground biomass - BGB); standard errors reflect error propagation.

	2001 Start (Bare)	2013 Gross	2013 Net	2016 Gross	2016 Net
Meadow area (km^2^)	0.096	5.79	5.79	6.86	6.86
AGB	0	−710 ± 14.8	−710 ± 14.8	−810 ± 17.6	−810 ± 17.6
Live BGB	0	−339 ± 30.0	−339 ± 30.0	−401 ± 33.2	−401 ± 33.2
Dead BGB	0	−857 ± 44.3	−857 ± 44.3	−1020 ± 52.5	−1020 ± 52.5
Sediment C_org_	−78 ± 6.29^a^	−13500 ± 792	−4150 ± 412	−20400 ± 3440	−7360 ± 1790
Total GHG Benefit			−6060		−9590
CH_4_	0.5 ± 0.20	385 ± 177	335 ± 156	611 ± 275	532 ± 249
N_2_O	1.5 ± 0.64	420 ± 152	264 ± 84.6	667 ± 243	420 ± 134
CO_2_ from CaCO_3_	3.8 ± 1.14	450 ± 137^b^	0^c^	623 ± 190^c^	0^c^
Total Emissions	5.7	1260	599	1780	952
**Net GHG benefit**			−5460		−8630

^a^background (i.e. baseline) stock within total seed plot area.

^b^The CO_2_ and CaCO_3_ gas exchange/reaction ratio may vary; we used 0.6, as discussed in the methods^26^.

^c^Note that we did not observe seagrass-enhanced CaCO_3_ burial in this system.

The average aboveground biomass standing stock over three years was 109 gdw m^−2^, equivalent to approximately 40.5 g C_org_ m^−2^. This reflects seasonal fluctuations that ranged from 330 g dry weight (gdw) m^−2^ in August (201.4 ± 29 g live plus 129.7 ± 15 g dead) to 38.5 gdw m^−2^ in March (19.58 ± 4.8 g live plus 18.86 ± 2.4 g dead) (see Supplement). All reported errors relate standard errors (SE), unless otherwise stated. The average aboveground biomass shoot^−1^ was 0.4 ± 0.07 gdw. Multiplying the average annual biomass per shoot by the interpolated average annual density values and integrating over the meadow area yielded an aboveground biomass standing stock of 710 t CO_2_ in 2013 and 810 t CO_2_ in 2016, due to meadow expansion. This standing stock is the average amount of C_org_ held in seagrass biomass throughout the year and is less than a third of the peak biomass in summer. Live belowground biomass ranged from 35.51 ± 7.3 gdw m^−2^ in January to 95.26 ± 13 gdw m^−2^ in August; the average annual live belowground biomass was 47.1 gdw m^−2^ (Supplement). Dead belowground biomass ranged from 91.03 ± 17 gdw m^−2^ in June 2016 to 131.91 ± 12 gdw m^−2^ in March, yielding an average, annual dead belowground biomass of 119 gdw m^−2^ (Supplement). Average, annual unit area estimates for live and dead belowground biomass were 16.0 and 40.4 g C_org_ m^−2^, respectively. Multiplied by the respective meadow areas, the combined belowground biomass stock sequestered 1,200 t CO_2_ in 2013 and 1,520 t CO_2_ in 2016.

Sediment C_org_ represented the largest sequestered carbon pool in the meadow in both 2013 and 2016, accounting for 68.5% of the total GHG benefit in 2013 and more than three-quarters of the total GHG benefit in 2016 (Table 3). Annual belowground biomass (live + dead) accounted for 14.7% of the total 2016 sequestered stock, and aboveground biomass represented 8.4%. Enhanced sediment C_org_ and the average, annual seagrass stock sequestered a combined 6,060 t CO_2_ in 2013 and 9,590 t CO_2_ in 2016 (Table 3).

The total, cumulative gross primary production (GPP) in the meadow from 2001–2013 was calculated to be 39,700 t CO_2_. By 2016, this estimate had increased to 84,900 t CO_2_, due to meadow expansion. Total, enhanced C_org_ sequestration was, therefore, 15.3% of cumulative GPP in 2013 and 11.3% in 2016.

### Enhanced GHG emissions and the net GHG benefit

**Figure 4:**
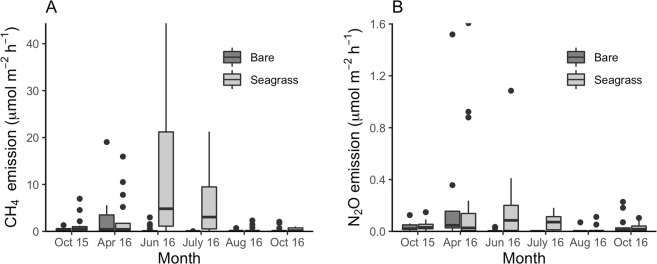
CH_4_ (**A**) and N_2_O (**B**) ebullition flux (μmol m^−2^ hr^−1^) box plots (quartiles) at sites (n = 10) by observation month (Oct. 2015–Oct. 2016) and by treatment (bare and seagrass). See Table 4 for log-likelihood ratio test results for assessing the treatment effect.

Seagrass presence significantly increased both the CH_4_ (χ^2^(1) = 13.1, *p* < 0.0003) and the N_2_O fluxes (χ^2^(1) = 8.46, *p* < 0.004) (Fig. 4A,B; Table 4). There was seasonal variation with seagrass presence*month interaction significant for both CH_4_ (χ^2^(10) = 36.4, *p* < 7.08e-5) and N_2_O release (χ^2^(10) = 35.8, *p* < 9.09e-5). The seagrass CH_4_ flux was highest in June, 15.9 ± 6.95 (SE) µmol CH_4_ m^−2^ hr^−1^ and lowest in August, 0.32 ± 0.22 (SE) µmol CH_4_ m^−2^ hr^−1^. The October 2016 flux was also low, 0.38 ± 0.06 (SE) µmol CH_4_ m^−2^ hr^−1^. The average bare site CH_4_ flux ranged from 3.37 ± 1.60 (SE) µmol CH_4_ m^−2^ hr^−1^ in April to 0.01 ± 0.007 (SE) µmol CH_4_ m^−2^ hr^−1^ in July. The average, annual enhanced CH_4_ flux was 0.70 ± 0.46 (SE) g CH_4_ m^−2^ yr^−1^. This represents the average, annual fluxes of 0.80 ± 0.53 (SE) g CH_4_ m^−2^ yr^−1^ from vegetated sites minus the average flux (0.10 ± 0.07 (SE) g CH_4_ m^−2^ yr^−1^) in bare sites (Fig. 4A; Table 1).

**Table 4:** Log-likelihood ratio test results for assessing a seagrass treatment effect (presence/absence) and a treatment*month interaction effect on benthic CH_4_ and N_2_O fluxes; rows relate the null model, reduced model, and full model for CH_4_ and N_2_O, respectively.

	Df^a^	logLik	deviance	χ^2^	Df^b^	Pr(>χ^2^)
CH_4_^(0.133) ~ 1 + (1|ID)	3	−9.25	18.50			
CH_4_^(0.133) ~ Treat + (1|ID)	4	−2.71	5.41	13.08	1	2.98E-04
CH_4_^(0.133) ~ Treat + Treat*Month + (1|ID)	14	15.51	−31.02	36.44	10	7.08E-05
N_2_O^(0.133) ~ 1 + (1|ID)	3	9.70	−19.39			
N_2_O^(0.133) ~ Treat + (1|ID)	4	13.92	−27.84	8.45	1	3.65E-03
N_2_O^(0.133) ~ Treat + Treat*Month + (1|ID)	14	31.83	−63.65	35.81	10	9.09E-05

^a^Mixed effects model degrees of freedom determined by lmer function (see Bates *et al*.^78^)

^b^Likelihood ratio test degrees of freedom (the difference between models used in each comparison).

**Figure 5:**
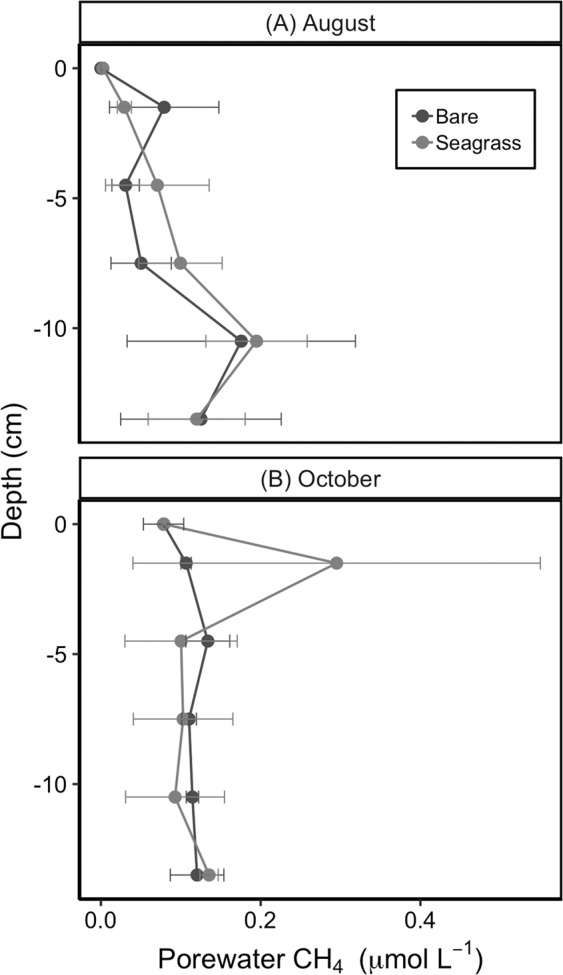
Porewater profile CH_4_ concentrations measured at bare and seagrass sites in August (**A**: site n = 6) and in October 2016 (**B**: site n = 4).

Bulk porewater CH_4_ concentrations measured at seagrass and bare sites in August and October yielded a negligible diffusive flux (Fig. 5). The highest average CH_4_ porewater concentration was 0.30 ± 0.25 µmol L^−1^ at 1.5 cm below the sediment water interface at seagrass sites in October. The highest average concentrations in August were observed at 10.5 cm below the sediment water interface, 0.18 ± 0.14 µmol L^−1^ at the bare sites and 0.19 ± 0.06 µmol L^−1^ at the seagrass sites (Fig. 5). Assuming a sediment diffusivity of 0.1 × 10^–4^ cm^2^ s^−1^ and using Fick’s first law of diffusion, a CH_4_ concentration of 0.02 nmol cm^−3^ gave a diffusive flux of -0.007 µmol m^−2^ hr^−1^. This flux was negligible compared to CH_4_ emissions captured in the water column and was therefore excluded from subsequent GHG accounting.

**Figure 6:**
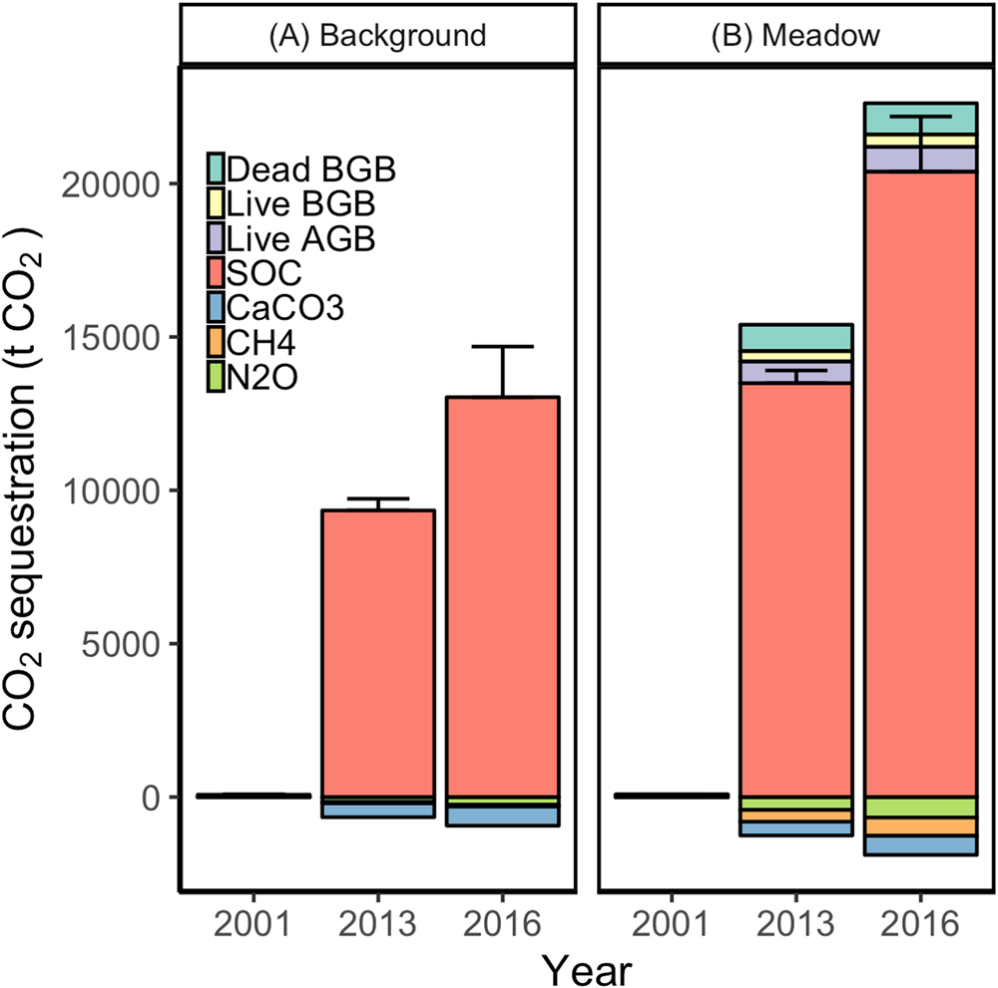
Cumulative background (**A**) and gross meadow (**B**) GHG stocks in the meadow areas over time; sequestration (i.e., GHG uptake from the atmosphere) in this figure is shown as positive, GHG release (i.e., a GHG flux to the atmosphere) is negative; CH_4_ and N_2_O quantities were standardized to CO_2_e; ‘CaCO_3_’ relates CO_2_ evasion attributable to CaCO_3_; background stocks were calculated by scaling average bare site values by total meadow area at each time step; net stock enhancement attributable to the meadow (see Table 3**)** can be calculated by subtracting the bare values (**A**) from equivalent gross meadow values (**B**); Error bars represent SE for the sediment C_org_ (SOC) stock.

Average N_2_O fluxes in the seagrass meadow ranged from 0.67 ± 0.42 (SE) in April to 0.01 ± 0.01 (SE) µmol N_2_O m^−2^ hr^−1^ in August. N_2_O fluxes were also lower at bare sites, ranging from 0.21 ± 0.14 (SE) in April to 0.001 ± 0.0004 (SE) µmol N_2_O m^−2^ hr^−1^ in July (Fig. 4B). The average, annual vegetated flux of 0.06 ± 0.04 (SE) g N_2_O m^−2^ yr^−1^ minus the average, annual bare flux of 0.02 ± 0.01 (SE) g N_2_O m^−2^ yr^−1^ yielded an enhanced flux of 0.04 ± 0.03 (SE) g N_2_O m^−2^ yr^−1^ (Table 2). Scaling the trace GHG fluxes by meadow area over time and by their 100-year global warming potentials^36^, meadow-enhanced CH_4_ and N_2_O fluxes released 530 and 420 t CO_2_e between 2001–2016, respectively (Table 3; Fig. 6).

We did not find a significant difference between average C_inorg_ concentrations by paired depth horizon in bare and seagrass sediment cores (*t* = -0.287, df = 13, *p* > 0.389). Inorganic carbon concentrations in the top 12 cm of the bed were similar throughout the meadow (site n = 16), averaging 0.11 ± 0.04 mg C_inorg_ cm^−3^. Scaling our average concentration from the top 6 cm of the bare site, 0.13 ± 0.04 mg C_inorg_ cm^−3^, by meadow area gave estimated CO_2_ emissions from CaCO_3_ formation of 450 t CO_2_ in 2013 and 623 t CO_2_ by 2016. However, the absence of a significant difference in CaCO_3_ between bare and seagrass sites meant that there was no net CO_2_ evasion attributable to the seagrass restoration (Table 3), so seagrass-enhanced CO_2_ evasion from CaCO_3_ between 2001–2016 was zero.

Integrating both stock changes and fluxes, this seagrass meadow restoration generated a net GHG benefit, which increased from 0.21 t C ha^−1^ yr^−1^ between 2001–2013 to 0.42 t C ha^−1^ yr^−1^ from 2013–2016, 12–15 years after restoration started.

## Discussion

By applying the VCS GHG accounting methodology for the first time to an actual seagrass restoration project^24^, this study confirms the generally accepted but essentially untested hypothesis that seagrass restoration results in net GHG removal from the atmosphere*—*a GHG offset benefit that can potentially finance restoration. We also found that seagrass presence increased both CH_4_ and N_2_O release, but these increases had a relatively small effect on the net GHG benefit. Although other studies have reported increases in gross seagrass bed sediment carbon concentrations following seagrass restoration (e.g.^9,12^,), these reports do not translate directly to an offset benefit^18^. As we demonstrate in this study, gross C_org_ stocks determined in previous studies overestimate the GHG offset benefit, because they do not account for background C_org_ sequestration that would occur in the absence of seagrass or GHG flux increases due to meadow restoration. All of these parameters must be known to determine the GHG offset benefit provided by seagrass restoration. This study also demonstrates the utility of the stock change approach for seagrass GHG offset accounting and addresses questions about stock change feasibility^18^.

### Seagrass-effects on CH_4_ and N_2_O release

The enhanced CH_4_ emissions reported here marginally exceeded the *de minimis* threshold, as defined by the VCS (<5%), reducing the total GHG benefit by 5.5% in 2013 and by 5.6% in 2016. By comparison, Rosentreter *et al*.^47^ estimated a 20.5% offset for methanogenesis in a tropical Australian mangrove forest. The enhanced N_2_O flux for the seagrass restoration was technically *de minimis*, 4.7% in 2013 and 4.4% in 2016. However, it is important to note that both seagrass trace gas fluxes reported here, 0.695 g CH_4_ m^−2^ yr^−1^ and 0.037 g N_2_O m^−2^ yr^−1^, exceeded the conservative general default factors for net benefit accounting, 0.56 g CH_4_ m^−2^ yr^−1^ (for salinities>20 ppt) and 0.016 g N_2_O m^−2^ yr^−1^ (Section 8.1.4.3.4 in^24^). These general default factors may, therefore, underestimate the magnitude of CH_4_ and N_2_O fluxes in other seagrass systems.

We observed considerable variability in CH_4_ and N_2_O fluxes at seagrass sites, especially during spring and summer months. More work is needed to understand site-specific drivers of CH_4_ and N_2_O production to better constrain annual fluxes^48^. This includes determining whether CH_4_ production varies with sediment C_org_ concentrations, whether CH_4_ and CO_2_ interactions affect CH_4_ release, and whether microbial community differences affect CH_4_ and N_2_O enhancement. We also note that using benthic chambers may have moderated release rates for both trace gases by inhibiting flow-induced efflux and that using experimentally cleared control sites, rather than bare sites outside the meadow, may have reduced the apparent seagrass enhancement effect. We advise other seagrass blue carbon studies to measure both trace gases directly, until a sufficient number of additional studies suggest conservative release rates for seagrass GHG accounting that are generally applicable.

### Identifying the net GHG benefit from seagrass restoration

Studies based on burial rates have suggested that seagrass meadows may sequester more carbon in soils than terrestrial forests^55^. The net sequestration rate based on sediment and plant stock changes and emissions of CH_4_ and N_2_O that we measured in this study, 0.42 t C ha^−1^ yr^−1^, is lower than the average rates for temperate and tropical forests, 2.6 and 5.3 t C ha^−1^ yr^−1^, respectively^51^, but generally agrees with the IPCC sequestration rate for seagrass systems, 0.43 t C ha^−1^ yr^−1^ ^51^. Similar studies in other systems may also support the use of this default factor, but we note several reasons why this default factor may not be an appropriate rate for all seagrass systems at all times. First, the IPCC rate is double the sequestration rate that we calculated for the first decade of our restoration, 0.21 t C ha^−1^ yr^−1^. Long-term research in this restored meadow has shown that it took about a decade for sediment carbon sequestration rates and plant biomass to be equivalent to natural meadows^12^. Second, sediment accretion may vary throughout the meadow. We assumed uniform sediment accretion, but actual accretion may be lower near the meadow edge, as evidenced by the grain size distribution and reported Reynolds stresses^56,57^ (see the Supplement). Third, our system has negligible carbonate, because the sediment in the region is siliciclastic, and there are no nearby coral reefs. We did not expect to find a significant difference in CaCO_3_ at seagrass and bare sites. This is not the case in other seagrass systems, where increased CO_2_ evasion may be significant (see the comparison between this system and others in Sadrene *et al*.^22^). Finally, the South Bay meadow also appears to be metabolically balanced on a decadal time scale, but studies in autotrophic systems may need to determine whether direct plant metabolism increases *p*CO_2_ and results in a CO_2_ flux back to the atmosphere^58,59^. These caveats point to areas where future research needs to be done to verify how generally the IPCC default factor applies to seagrass ecosystems worldwide.

The stock change approach indicates that the carbon sequestration rate for this meadow is increasing but that net CO_2_ sequestration as a percentage of meadow-wide community GPP may be declining with meadow age. Cumulative GPP increased by 114% between 2013 and 2016, due largely to meadow expansion, but the enhanced sequestered stock only increased by 78% over this period. The fraction of GPP that is sequestered may increase over time if the meadow stops expanding and GPP reaches a long-term steady state. Recent work at this site has shown that GPP initially exceeded respiration in this meadow but later reached equivalence^59,60^, a finding that may pertain to eelgrass systems generally^61^. Studies need to determine whether carbon sequestration as a percentage of GPP changes over time in other systems, including those that appear to be net autotrophic^30^, and whether seagrass offset benefits continue to accumulate indefinitely.

Given that measuring sediment C_org_ stock changes in a seagrass system is feasible, we recommend using this method to calculate seagrass net GHG benefits to avoid issues associated with using burial fluxes for this purpose^18^. Use of ^210^Pb dating to calculate sedimentation rates in seagrass systems has been criticized where relatively short (decadal) time scales are addressed and where bioturbation could disturb sediment profiles^18^. A recent study used surface elevation tables (SET) to compare changes in surface elevation between bare and seagrass sites over short (<1 yr) time scales^62^, but the SETs and marker horizons used widely in salt marshes are generally problematic in seagrass meadows. Subtidal currents re-suspend surface sediments, scouring occurs around vertical objects, including SET pins, and the high-water content of surface sediment makes precise (mm-scale) measurements of surface elevation difficult^63^. Burial rate sequestration estimates also assume that surface deposition is the primary vector for transferring C_org_ to the sediment, but we observed considerable C_org_ accumulation within the bed. This may be due to sediment C_org_ accumulation from root C_org_ exudates or from increased preservation of benthic microalgae migrating up and down within the sediment^64^. The sediment C_org_ stock increase that we observed, 874 t C_org_, exceeded the increase we would have estimated by scaling the Greiner *et al*.^12^ surface burial flux reported for this system by meadow area and by the three-year time period, 755 t C_org_. However, we also observed sediment C_org_ declines in 2016 at particular sites, which affected the 2016 sediment C_org_ spatial distribution (Fig. 3). Random disturbance events will likely affect long-term (i.e. decadal) sediment C_org_ accumulation rates by periodically removing sequestered sediment C_org_ stocks. A stock change approach captures these changes. Burial flux rates derived from dated sediment cores may need to be reconsidered, given the magnitude of the within bed C_org_ accumulation that we observed.

Individual seagrass projects should also take care to avoid overestimating the GHG offset benefit by failing to account for allochthonous C_org_. The VCS carbon-offset protocol conservatively requires that carbon fixed outside the project area (allochthonous carbon) be excluded from the GHG offset benefit, because this cannot be unequivocally attributed to the seagrass restoration project^18,24^. We conservatively deducted the background C_org_ concentration from the entire seagrass C_org_ profile to account for possible deposition of allochthonous carbon (see Fig. 1 and the Supplement). Including all of the sediment C_org_ in the accreted part of the South Bay bed would have almost doubled the apparent project benefit to 10.1 K t CO_2_e in 2013 and 17.2 K t CO_2_e in 2016.

### Offset-credit finance as an incentive for seagrass restoration

Had this restoration project been able to apply for VCS offset-credits in 2001, it would now receive up to 8,630 credits. The actual allocation of credits would be slightly lower to account for CO_2_ emissions from project activities (i.e. travel to restoration sites, etc.) and ‘buffer pool’ set aside credits to account for the risk of GHG offset gain reversals^24^. Investors do not typically consider GHG offset projects viable unless they sequester at least 50,000 tCO_2_e over the project lifetime (typically 30 years)^65^. Reaching 50,000 credits by 2031 would require a further increase in the C sequestration rate by this meadow. Future work, including repeated carbon stock change measurements and bed accretion measurements, will be necessary to determine whether the sequestration rate continues to increase.

Given current market prices, carbon offset-credits currently provide a marginal incentive for seagrass restoration. At a price of $10 ton^−1^, offset-credits would finance approximately 10% of the approximately $800 K South Bay restoration cost^17,66^. Fully financing a seagrass restoration project with a unit cost equivalent to this South Bay *Z. marina* restoration would require a voluntary offset price greater than $95 per MtCO_2_e. This cost-benefit comparison excludes project development costs, which may exceed $100 K, and net present value discounting. We note that the carbon burial rates measured in South Bay are on the low end of those documented for other seagrass meadows globally^5^. Other species and locations may generate larger sediment C_org_ stocks than we measured for *Z. marina* over time (e.g.^67)^. However, the South Bay restoration was accomplished at a unit cost of only $1,200 ha^−1^ ^17^, and the range for other seagrass projects is $1,900–4,000,000 ha^−1^ ^54^.

Rather than rely solely on carbon offset-credits to finance meadow restoration, coastal managers should think holistically about the other values that seagrass systems provide, including fisheries support, nutrient removal, and reduced marsh erosion, among other services. Quantifying these values, even absent markets for co-benefit ‘credits,’ would provide further incentive for seagrass restoration, in addition to carbon sequestration.

## Methods

### Study area

We measured all of the parameters required by the VCS methodology to quantify the GHG offset benefit from the *Z. marina* restoration in South Bay, VA^24^. The restoration history^68^, project cost^17^, sediment C_org_ stock enhancement^12,57,69^, and net ecosystem metabolism^58–60^ of this meadow have been documented and provide a baseline for stock-change assessment. The South Bay meadow area is shallow, with an average depth at mean sea level of 0.76 ± 0.28 (SD) m, and oligotrophic, with low nutrient loading (Fig. 2)^57^. For additional background on the Virginia Coast Reserve Long-Term Ecological Research eelgrass restoration, including reseeding methods, see Orth and McGlathery^68^ and other studies in the *Marine Ecology Progress Series* v. 448^69,70^.

### Sediment C_org_ stock enhancement

Meadow sediment C_org_ stock enhancement was determined for both 2013 and 2016 by subtracting baseline sediment (i.e., bare) C_org_ stocks from the gross stocks measured within the meadow (Fig. 1). C_org_ is generally present in subtidal sediment without seagrass meadows, and this background C_org_ should not be attributed to a seagrass restoration project. The restored meadow was already in existence when we began sampling in 2013, so time = 0 values at sites within the meadow were not available. The sediment C_org_ baseline scenario (the Emmer *et al*.^24^ ‘without project’ scenario) that would represent pre-restoration (time = 0) was, therefore, established by measuring C_org_ concentrations at bare control sites outside the meadow. The average C_org_ concentration in cores collected at four bare sites by Greiner *et al*.^12^ in 2011 and by Oreska *et al*.^57,64^ in 2013 and in 2014 was 3.67 ± 0.55 (SE) mg C_org_ cm^−3^ (see Supplement). We verified that this background concentration remained unchanged by collecting new, replicate cores (n = 4) at two of these bare sites in 2016. We deducted this average background sediment C_org_ concentration from the sediment C_org_ concentrations measured within the meadow in 2013 and in 2016 to identify the C_org_ attributable to the seagrass restoration (Fig. 1). This is in accordance with the stock change assessment recommended by the VCS methodology^24^.

We assessed C_org_ changes at sites within the meadow in 2016 by resampling 16 randomly-selected meadow sites first sampled by Oreska *et al*.^57^ in 2013 (the ‘with project’ scenario). Four 12-cm long, 2.7 cm diameter cores were collected at each site and subdivided into 3-cm intervals. Macroscopic roots and rhizomes were removed from each sample manually, using tweezers. Note that belowground biomass (BGB) was quantified separately, as described in the following section, to avoid double counting. All sediment samples were prepared according to methods used previously in this system^12,57,64^. We measured %C on a Thermo Scientific Flash 2000 Organic Element Analyzer; %C_org_ was determined by subtracting %C_inorg_, which we determined using element analysis of samples ashed at 500 °C for six hours^71^. The element analyzer average percent error was 0.48%, based on analysis of lab standards.

Allochthonous C_org_ may be deposited within the bed due to bed accretion (Fig. 1). Rather than deduct an arbitrary ‘allochthonous compensation factor’ from the meadow sediment C_org_ stock^72,73^, we accounted for allochthonous C_org_ that could have been deposited in the baseline scenario by deducting the bare site sediment C_org_ average from the entire meadow carbon profile, including the part of the sediment profile that may have resulted from accretion facilitated by the meadow (see Fig. 1 and the Supplement for more explanation).

Total, meadow-enhanced sediment C_org_ stocks in 2013 and in 2016 were quantified by interpolating the average 2013 and 2016 sediment C_org_ enhancement at each site in ArcGIS 10.2 Geostatistical Analyst using Ordinary Kriging^74^. We fitted stable, circular, spherical, Guassian, and exponential semivariogram models to each dataset and selected the sediment C_org_ distribution maps with the lowest root mean square errors (Supplement). The 2013 data was best fit using a circular model, the equivalent 2016 data was best fit using a Gaussian model, and the uppermost 2-cm interval in 2016, which may be the result of accretion and is shown separately in Fig. 3, was best fit using an exponential model.

### Biomass CO_2_ sequestration

The carbon sequestered in seagrass tissue is periodically lost to export, herbivory, and decomposition, so we calculated and reported the average, annual standing biomass stock based on seasonal measurements from 2014–2016 (see Supplement). This represents a running average that reflects periodic export and other fluctuations, rather than peak observed biomass. This is the same general approach that reforestation GHG offset projects use to address the cyclical harvest and replanting of aboveground biomass (AGB), and it is permitted for seagrass GHG accounting^24^. Shoot densities ranged from approximately 250 to 617 shoots m^−2^ in South Bay due to seasonal thinning and export, and biomass ranged from 0.26 to 0.781 gdw shoot^−1^. We accounted for variability in AGB using existing density measurements (shoots m^−2^) taken at sites throughout this meadow over time to account for seasonal changes^57,75^. The average density over the course of a year was approximately half of the peak density observed during July (48%)^57,75^

We quantified average AGB per shoot and BGB by collecting additional replicate (n ≥ 4) 15.2-cm diameter biomass cores seasonally from June 2014 to June 2016 to a depth of 15 cm at five central meadow sites (see Supplement), following methods employed by past studies in this system^69,76^. We also collected biomass cores (n ≥ 3) at four additional, systematically located sites during the summer of 2016 (see Supplement). Samples were sieved using a 1-mm mesh, separated the same day into live and dead fractions, and then dried to a constant weight at 60 °C. Biomass data—both live and dead—was averaged by site and then by month to generate seasonal averages, which were used to calculate the average, annual standing stocks. The average, annual shoot densities were multiplied by the average biomass shoot^−1^, 0.41 ± 0.09 gdw shoot^−1^ (this study), and by 37.1% C gdw^−1^ biomass^76^. The resulting aboveground biomass values (C_org_ m^−2^) were interpolated using Ordinary Kriging in ArcGIS 10.2 Geostatistical Analyst and a Gaussian semivariogram to generate average, annual AGB stocks for the 2013 and 2016 meadow extents. Average live and dead BGB values (g m^−2^) were multiplied by the average C_org_ fraction in belowground biomass, 33.8% C_org_ gdw^−1^ biomass^77^, and scaled by the 2013 and 2016 meadow areas to generate C_org_ stocks.

### GHG fluxes

We deployed clear plastic, bell-shaped benthic chambers over vegetated and experimentally cleared 2 m x 2 m bare plots at the five central meadow sites to identify changes in benthic CH_4_ and N_2_O fluxes attributable to *Z. marina* presence. Each chamber sat on the sediment surface, covering a 0.046 m^2^ circular area and enclosing a 10.5 L volume. Comparing fluxes at cleared, central meadow plots allowed us to control for confounding factors at bare sites outside of the meadow. These areas are generally deeper with more sand-sized sediment and experience greater Reynolds stresses, because of area geomorphology^56^, factors that may affect sediment:water gas exchange. We cleared the bare plots during spring 2015, installed plastic lawn edging to a depth of 8 cm to prevent seagrass rhizome re-colonization, and allowed plots to equilibrate for five months. Comparing seagrass and cleared bare plots to assess a seagrass enhancement effect on CH_4_ and N_2_O was conservative, because some seagrass BGB potentially remained at the cleared plots and may have contributed to microbial production of these trace gases. Eight chambers were deployed at each site during each observation, four replicates over seagrass and four over bare sediment. Every deployment exactly bracketed low tide, such that gas accumulation time captured equal parts falling- and rising-tide. Deployment durations ranged from 1 to 5 hours. Trace gases were collected on multiple days per month in October 2015, April 2016, June 2016, July 2016, August 2016, and October 2016. Using chambers allowed us to conduct a controlled experiment *in situ* to test for a seagrass presence effect, but we acknowledged that using benthic chambers may have introduced container effects that affected release rates, including the elimination of hydrodynamic flow-induced efflux.

The gas that collected in each chamber was syringe extracted and injected into an exetainer filled with 12 ml N_2_ and 0.2 ml 0.01 M ZnC_4_H_6_O_4_ to prevent microbial activity resulting from the syringe transfer. The total gas volume collected within each chamber was noted and used to calculate the gas flux as a function of time and bed surface area. We also measured bulk CH_4_ concentrations in replicate porewater samples collected at bare and vegetated sites in August (site n = 6) and October (site n = 4) 2016 to determine the magnitude of the diffusion flux relative to the ebullition flux. We extracted 7 ml of porewater through mini-piezometers (inner diameter 1.8 mm) at 3-cm intervals, from 1.5 cm down to 13.5 cm. The water samples were syringe injected into exetainers filled with 12 ml N_2_ and fixed with 0.2 ml ZnCl_2_. The diffusive flux was calculated using Fick’s first law of diffusion:

1 $${\rm{Flux}}=-\,{\rm{DsdC}}/{\rm{dx}}$$

where the sediment diffusivity, Ds, was assumed to be 0.1 × 10^–4^ cm^2^ s^−1^.

All exetainer samples were analyzed on a Varian 450-Gas Chromatograph with a Bruker GC/MS workstation at the Smithsonian Environmental Research Center. We determined sample CH_4_ and N_2_O concentrations using onsite standards and corrected for differences in atmospheric temperature and pressure during each GC analysis. Standard curve R^2^ values ranged from 0.992 to 0.996.

We tested for an effect of seagrass presence on CH_4_ and N_2_O fluxes using linear mixed effect models in R^78,79^. Replicate results were averaged by site. Seagrass presence/absence and month were treated as fixed effects; individual sites were randomly selected. Tests were run on each GHG dataset using the lmer function (lme4 package version 1.1–14). We expected to find increased GHG fluxes attributable to seagrass presence, as well as a seagrass*month interaction effect. Both the CH_4_ and N_2_O datasets required transformation due to heteroskedasticity and the presence of outliers. The optimal transformation (identified using the optim.boxcox function in the boxcoxmix package version 0.14) for the averaged data was λ = 0.133 (Maximum log-likelihood = −77.608). Model *p*-values were obtained from likelihood ratio tests on the full model and a reduced model without the fixed effects. Average, annual seagrass and bare CH_4_ and N_2_O fluxes were determined by first averaging fluxes by season and then averaging the seasonal averages. Note that the early June observations were included as spring values and that we conservatively reported 9-month averages. The difference between seagrass and bare values represented the net fluxes attributable to seagrass presence. All statistics were calculated in R (R stats package version 3.4.2)^79^.

CO_2_ evasion attributable to C_inorg_ was estimated by multiplying the C_inorg_ stock by a CO_2_ and CaCO_3_ gas exchange/reaction ratio of 0.6, following Howard *et al*.^26^. We determined whether or not seagrass presence increased C_inorg_ concentrations by running a paired *t*-test on average, depth-calibrated C_inorg_ concentrations from 20-cm cores collected at a representative meadow site and a representative bare site in this system.

### Net GHG benefit accounting

Total meadow CO_2_ sequestration was calculated for both 2013 and 2016 by summing the above- and belowground biomass (both live and dead) and meadow-enhanced sediment C_org_ stocks measured in each year. Cumulative, enhanced CH_4_ and N_2_O emissions attributable to the meadow were estimated by multiplying the average enhanced (i.e., net) fluxes (g m^−2^ yr^−1^) by meadow area over time. Meadow area changes were calculated in ArcGIS 10.2 by georeferencing the Virginia Institute of Marine Science aerial photographs for every year after initial reseeding in 2001 and delineating the meadow perimeter^74,80^. Meadow area was interpolated for the three years where photographs were unavailable. These cumulative, net GHG emissions calculated for 2013 and for 2016 were subtracted from the respective meadow-enhanced CO_2_ sequestration results to determine the net GHG benefit in each year (note that seagrass-enhanced CO_2_ emissions from CaCO_3_ were not observed).

We compared the total meadow sequestration in 2013 and in 2016 with the total, cumulative GPP within the meadow in each of those years to estimate the percentage of total GPP sequestered by the meadow. Cumulative GPP was estimated as a function of shoot density and meadow area. The relationship between meadow age and density was determined by fitting a polynomial regression to existing data from this meadow collected as part of the annual VCR-LTER seagrass survey^81^. This relationship was observed by Berger *et al*.^59^ to be:

2 $${\rm{Y}}=-\,0.678{{\rm{x}}}^{3}+13.058{{\rm{x}}}^{2}\,\mbox{--}\,9.42{\rm{x}}$$

where Y was shoot density in shoots m^−2^, and x was the meadow age in years (R^2^ = 0.91). GPP was calculated using the following relationship observed in this meadow by Berger *et al*.^59^:

3 $${\rm{Y}}=48.955+0.304{\rm{x}}$$

where x was density (shoots m^−2^) and Y was GPP in mmol O_2_ m^−2^ d^−1^ (R^2^ = 0.69). Calculated GPP values for meadow areas of different age were summed and integrated over time to generate cumulative values.

## Supplementary information

Supplementary information.

## Data Availability

Data reported and analyzed in this study is available in the Supplement and on the LTER Network Data Portal (https://portal.lternet.edu/nis/home.jsp).
