# Supplementary material for: The greenhouse gas offset potential from seagrass restoration
Source: Sci Rep. 2020 Apr 30;10:7325. doi: 10.1038/s41598-020-64094-1 (PMC7193639; doi:10.1038/s41598-020-64094-1)
Supplement: Supplementary file 1 — Supplementary information. [file 41598_2020_64094_MOESM1_ESM.docx]

**The greenhouse gas offset potential from seagrass restoration – Supplement**

Matthew P. J. Oreska, Karen J. McGlathery, Lillian R. Aoki, Amélie C. Berger, Peter Berg, Lindsay Mullins

**Quantifying Seagrass-Enhanced Sediment C_org_ and Accounting for Bed Accretion:**

The VCS methodology for determining the greenhouse gas offset potential of seagrass restoration tasks projects with measuring C_org_ to a sediment depth where the seagrass sediment C_org_ concentration becomes “indistinguishable” from the concentration observed at bare sites—the reference plane (Emmer et al.^[[1]](#endnote-1)^). Some studies of seagrass blue carbon report large carbon stocks based on measurements of sediment C_org_ to an arbitrary depth, often 1 m (e.g., Fourqurean et al.^[[2]](#endnote-2)^); however, this approach likely overestimates the sediment C_org_ specifically attributable to the seagrass meadow presence (see Johannessen and Macdonald^[[3]](#endnote-3)^). Most studies to date have relied on ^210^Pb dating of sediment profiles to determine bed volume change and C_org_ accumulation rates (Saderne et al.^[[4]](#endnote-4)^); however, surface sediments often are bioturbated, and ^210^Pb dates may not provide reliable estimates for short (decadal) time scales (Johannessen and Macdonald^3^). Most subtidal seagrass beds are too dynamic for surface elevation tables or marker horizons, because currents cause sediment scouring around the base of vertical structures like surface elevation table pins, and the high water content of surface sediments makes precise (mm-scale) measurements of surface elevation difficult (Lefebvre et al.^[[5]](#endnote-5)^).

We avoid these complications and rely on an alternate approach of repeated carbon stock change measurements in sediment profiles relative to a baseline scenario in bare sediments. This approach was suggested by Oreska et al.^[[6]](#endnote-6)^ as a method for determining the seagrass-enhancement of sediment C_org_ by a restored meadow, as recommended by the VCS methodology (Emmer et al.^1^).

We compared 20 cm sediment profiles collected from bare and seagrass sites in 2011 by Greiner et al.^[[7]](#endnote-7)^ and in 2014 by Oreska et al.^[[8]](#endnote-8)^ to identify the baseline scenario. For each set of profiles, we determined the depth at which the seagrass C_org_ concentration was equivalent to the bare C_org_. This occurred at a depth of approximately 12 cm within the seagrass cores. Sediments below these depths were below the influence of the restoration and showed no relative enhancement of C_org_ due to seagrass presence. We quantified the seagrass enhancement of C_org_ by subtracting the average background C_org_ (dark grey area in Figure 1 in the main text) from the C_org_ profile measured in seagrass sediments. The background C_org_ concentration was the average concentration within the top 6 cm of the bed at bare sites, 3.67 ± 0.55 (SE) mg C_org_ cm^-3^, a value that was fairly consistent across sites and over time, as evident from cores collected at bare sites in 2011, 2013, 2014, and 2016. Note that this concentration happens to be equivalent to the vertical part of the average 2011 meadow profile that is shown in Figure 1. We subtracted this background C_org_ concentration before integrating C_org_ by depth, in order to determine the seagrass-enhancement of C_org_ (light grey area in Figure 1). Deducting a single, average background concentration from the entire seagrass C_org_ profile allowed us to conservatively account for possible allochthonous C_org_ in the accreted part of the bed (see below). Finally, we interpolated C_org_ values from sites throughout the meadow using kriging and integrated over the meadow extent to find the meadow-wide enhanced C_org_ stock. Our repeated measurements of C_org_ in 2013 and 2016 (see Methods) enabled us to calculate the increase in the sediment C_org_ stock throughout the meadow over time.

**Accounting for Allochthonous C_org_:**

Non-seagrass carbon sources contribute more than half of the sediment C_org_ sequestered within this seagrass bed; however, most of this non-seagrass C_org_ was likely fixed in situ by benthic microalgae, and its preservation appears to be attributable to the meadow (Oreska et al.^8^). Only the percentage attributable to *Spartina alterniflora*, 10%, is definitively allochthonous, and this fraction is observed at sites both inside and outside of the meadow (Oreska et al.^8^). We note that the background C_org_ concentration measured at bare sites exceeds the amount attributable solely to *S. alterniflora* and, therefore, includes refractory organic carbon from other sources that would be sequestered absent the meadow. Rather than deducting the background concentration from the part of the seagrass bed that is stratigraphically equivalent to bare sites and just the *S. alterniflora* fraction from the accreted part of the bed, we conservatively deducted the background concentration (dark grey area in Figure 1 in the main text) from the entire C_org_ profile measured at meadow sites to identify the seagrass-enhanced C_org_ stock (light gray area in Figure 1). By deducting the background fraction from the accreted part of the bed, we also avoid including any allochthonous C_org_ in the GHG offset benefit. The meadow-enhanced C_org_ stock only corresponded to the light gray fraction in Figure 1. See Oreska et al.^6^ for additional explanation of this approach for deducting allochthonous C_org_.

**South Bay, Virginia, Eelgrass Biomass:**

The following new biomass data from the restored *Zostera marina* (eelgrass) meadow in South Bay, Virginia, U.S.A., was combined with existing data from this restored meadow to generate the meadow-scale above- (AGB) and belowground biomass (BGB) stock estimates presented in this study:

**Table S1. Seasonal aboveground biomass (AGB) data (g m^-2^) at mid-meadow sites.**

|  | Fraction | ABG | SD | VAR | n | SE |
| --- | --- | --- | --- | --- | --- | --- |
| Jun 2014 | Live | 144.50 | 52.88 | 2796.29 | 7 | 19.99 |
|  | Dead | 36.29 | 13.40 | 179.52 | 5 | 5.99 |
| Aug 2014 | Live | 201.42 | 64.27 | 4130.78 | 5 | 28.74 |
|  | Dead | 129.71 | 33.01 | 1089.70 | 5 | 14.76 |
| Oct 2014 | Live | 65.11 | 8.77 | 76.99 | 4 | 4.39 |
|  | Dead | 116.19 | 41.43 | 1716.09 | 4 | 20.71 |
| Jan 2015 | Live | 18.99 | 6.72 | 45.10 | 6 | 2.74 |
|  | Dead | 20.64 | 10.24 | 104.79 | 4 | 5.12 |
| Mar 2015 | Live | 19.58 | 10.66 | 113.58 | 5 | 4.77 |
|  | Dead | 18.86 | 5.42 | 29.33 | 5 | 2.42 |
| Apr 2015 | Live | 34.71 | 20.03 | 401.08 | 5 | 8.96 |
|  | Dead | 19.48 | 2.25 | 5.06 | 5 | 1.01 |
| Jun 2015 | Live | 50.48 | 33.09 | 1094.89 | 6 | 13.51 |
|  | Dead | 21.06 | 14.62 | 213.75 | 6 | 5.97 |
| Jun 2016 | Live | 86.04 | 50.57 | 2557.18 | 5 | 22.61 |
|  | Dead | 13.43 | 5.55 | 30.79 | 5 | 2.48 |

**Table S2. Seasonal belowground biomass (BGB) data (g m^-2^) at mid-meadow sites.**

|  | Fraction | BGB | SD | VAR | n | SE |
| --- | --- | --- | --- | --- | --- | --- |
| Jun 2014 | Live | 73.89 | 13.34 | 177.97 | 5 | 5.97 |
|  | Dead | 115.05 | 17.36 | 301.35 | 5 | 7.76 |
| Aug 2014 | Live | 95.26 | 28.76 | 827.30 | 5 | 12.86 |
|  | Dead | 112.66 | 27.83 | 774.46 | 5 | 12.45 |
| Oct 2014 | Live | 51.51 | 15.80 | 249.65 | 4 | 7.90 |
|  | Dead | 126.63 | 34.93 | 1220.29 | 4 | 17.47 |
| Jan 2015 | Live | 35.51 | 14.63 | 214.12 | 4 | 7.32 |
|  | Dead | 128.08 | 46.47 | 2159.64 | 4 | 23.24 |
| Mar 2015 | Live | 30.39 | 18.90 | 357.14 | 5 | 8.45 |
|  | Dead | 131.91 | 26.12 | 682.39 | 5 | 11.68 |
| Apr 2015 | Live | 44.44 | 25.81 | 666.31 | 5 | 11.54 |
|  | Dead | 91.19 | 18.23 | 332.28 | 5 | 8.15 |
| Jun 2015 | Live | 55.05 | 22.50 | 506.06 | 6 | 9.18 |
|  | Dead | 121.32 | 36.58 | 1338.35 | 6 | 14.94 |
| Jun 2016 | Live | 30.96 | 16.84 | 283.43 | 5 | 7.53 |
|  | Dead | 91.03 | 37.76 | 1426.06 | 5 | 16.89 |

**Table S3. Aboveground (AGB) and belowground (BGB: live + dead) biomass at mid-meadow and selected meadow-scale sites (g shoot^-1^)**

|  |  | AGB Avg | AGB SE | BGB Avg | BGB SE |
| --- | --- | --- | --- | --- | --- |
| Spatially Distributed Sites^A^ | Site 5 | 0.48 | 0.02 | 1.38 | 0.48 |
|  | Site 11 | 0.19 | 0.03 | 0.57 | 0.11 |
|  | Site 3 | 0.88 | 0.17 | 1.72 | 0.81 |
|  | Site 6 | 0.56 | 0.04 | 1.85 | 0.49 |
| Central Meadow Monitoring  Sites^B^ | SB105 | 0.33 | 0.21 | 0.90 | 0.30 |
|  | SB106 | 0.35 | 0.14 | 1.50 | 1.09 |
|  | SB128 | 0.11 | 0.02 | 0.81 | 0.49 |
|  | SB148 | 0.42 | 0.15 | 0.60 | 0.15 |
|  | SB152 | 0.35 | 0.01 | 0.30 | 0.02 |

^A^ Site locations from Oreska et al.^8^

^B^ Site locations available from McGlathery^[[9]](#endnote-9)^

This new data augmented the following publically available data:

The meadow-scale density distribution (shoots m^-2^) in the South Bay meadow was documented by Oreska et al.^[[10]](#endnote-10)^. Thomas^[[11]](#endnote-11)^ measured seasonal changes in density within the South Bay meadow. Biomass (g m^-2^) and canopy height (cm) data were provided by McGlathery^[[12]](#endnote-12)^. Average C_org_ content (C_org_ g^-1^ biomass) of seagrass biomass in the South Bay meadow was obtained from McGlathery^[[13]](#endnote-13)^. Interannual changes in density within the South Bay meadow were obtained from McGlathery^[[14]](#endnote-14)^.

**South Bay, Virginia, CH_4_ and N_2_O Flux Data:**

**Table S4. CH_4_ and N_2_O observation data from South Bay meadow sites (n.d. = no data).**

| Treatment | Site^A^ | Date | Time (hr) | Pressure (Hg) | Temp (°C) | Sal (ppt) | Density (shoot/m^2^) | CH_4_ flux (umol/m^2^/hr) | N_2_O flux (umol/m^2^/hr) |
| --- | --- | --- | --- | --- | --- | --- | --- | --- | --- |
| Seagrass | 128 | 10/9/15 | 2.1 | 30.135 | 22 | n.d. | 3 | 0.328 | 0.021 |
| Seagrass | 128 | 10/9/15 | 2.1 | 30.135 | 22 | n.d. | 3 | 0.310 | 0.029 |
| Seagrass | 128 | 10/9/15 | 2.1 | 30.135 | 22 | n.d. | 3 | 1.223 | 0.044 |
| Seagrass | 128 | 10/9/15 | 2.1 | 30.135 | 22 | n.d. | 3 | 2.166 | 0.078 |
| Seagrass | 152 | 10/9/15 | 1.0 | 30.135 | 21.2 | n.d. | n.d. | 6.955 | 0.148 |
| Seagrass | 152 | 10/9/15 | 1.0 | 30.135 | 21.2 | n.d. | n.d. | 0.408 | 0.030 |
| Seagrass | 152 | 10/9/15 | 1.0 | 30.135 | 21.2 | n.d. | n.d. | 4.556 | 0.000 |
| Seagrass | 152 | 10/9/15 | 1.0 | 30.135 | 21.2 | n.d. | n.d. | 0.686 | 0.012 |
| Seagrass | 105 | 10/11/15 | 2.5 | 30.135 | 20.2 | 33.5 | 5 | 0.551 | 0.047 |
| Seagrass | 105 | 10/11/15 | 2.5 | 30.135 | 20.2 | 33.5 | 5 | 0.568 | 0.047 |
| Seagrass | 105 | 10/11/15 | 2.5 | 30.135 | 20.2 | 33.5 | 5 | 0.213 | 0.028 |
| Seagrass | 105 | 10/11/15 | 2.5 | 30.135 | 20.2 | 33.5 | 5 | 0.566 | 0.093 |
| Bare | 105 | 10/11/15 | 2.5 | 30.135 | 20.2 | 33.5 | 0 | 0.506 | 0.046 |
| Bare | 105 | 10/11/15 | 2.5 | 30.135 | 20.2 | 33.5 | 0 | 1.324 | 0.126 |
| Bare | 105 | 10/11/15 | 2.5 | 30.135 | 20.2 | 33.5 | 0 | 0.103 | 0.015 |
| Bare | 105 | 10/11/15 | 2.5 | 30.135 | 20.2 | 33.5 | 0 | 0.230 | 0.016 |
| Seagrass | 128 | 10/11/15 | 2.0 | 30.135 | 20.1 | 33.5 | 3 | 0.945 | 0.079 |
| Seagrass | 128 | 10/11/15 | 2.0 | 30.135 | 20.1 | 33.5 | 3 | 0.245 | 0.018 |
| Seagrass | 128 | 10/11/15 | 2.0 | 30.135 | 20.1 | 33.5 | 3 | 0.286 | 0.032 |
| Seagrass | 128 | 10/11/15 | 2.0 | 30.135 | 20.1 | 33.5 | 3 | 0.091 | 0.013 |
| Bare | 128 | 10/11/15 | 2.0 | 30.135 | 20.1 | 33.5 | 0 | 0.300 | 0.025 |
| Bare | 128 | 10/11/15 | 2.0 | 30.135 | 20.1 | 33.5 | 0 | 0.858 | 0.062 |
| Bare | 128 | 10/11/15 | 2.0 | 29.9525 | 20.1 | 33.5 | 0 | 0.224 | 0.175 |
| Bare | 128 | 10/11/15 | 2.0 | 30.135 | 20.1 | 33.5 | 0 | 0.246 | 0.012 |
| Seagrass | 128 | 4/20/16 | 1.7 | 29.87 | 15.6 | 31.7 | 3.4 | 1.814 | 0.122 |
| Seagrass | 128 | 4/20/16 | 1.7 | 29.87 | 15.6 | 31.7 | 3.4 | 0.332 | 0.003 |
| Seagrass | 128 | 4/20/16 | 1.7 | 29.87 | 15.6 | 31.7 | 3.4 | 0.000 | 0.000 |
| Seagrass | 128 | 4/20/16 | 1.7 | 29.87 | 15.6 | 31.7 | 3.4 | 0.090 | 0.024 |
| Seagrass | 105 | 4/20/16 | 1.7 | 29.87 | 15.6 | 31.7 | 5.4 | 150.984 | 6.870 |
| Seagrass | 105 | 4/20/16 | 1.7 | 29.87 | 15.6 | 31.7 | 5.4 | 0.018 | 0.000 |
| Seagrass | 105 | 4/20/16 | 1.7 | 30.135 | 15.6 | 31.7 | 5.4 | 0.000 | 0.000 |
| Seagrass | 105 | 4/20/16 | 1.7 | 30.135 | 15.6 | 31.7 | 5.4 | n.d. | n.d. |
| Bare | 105 | 4/20/16 | 1.8 | 29.8075 | 15.6 | 31.7 | 0 | 0.349 | 0.038 |
| Bare | 105 | 4/20/16 | 1.8 | 29.87 | 15.6 | 31.7 | 0 | 19.037 | 0.162 |
| Bare | 105 | 4/20/16 | 1.8 | 29.87 | 15.6 | 31.7 | 0 | 0.049 | 0.023 |
| Bare | 105 | 4/20/16 | 1.8 | 30.135 | 15.6 | 31.7 | 0 | n.d. | n.d. |
| Bare | 128 | 4/20/16 | 1.7 | 29.87 | 15.6 | 31.7 | 0 | 5.584 | 1.519 |
| Bare | 128 | 4/20/16 | 1.7 | 29.87 | 15.6 | 31.7 | 0 | 3.646 | 0.357 |
| Bare | 128 | 4/20/16 | 1.7 | 29.87 | 15.6 | 31.7 | 0 | 0.348 | 0.037 |
| Bare | 128 | 4/20/16 | 1.7 | 29.87 | 15.6 | 31.7 | 0 | 0.068 | 0.014 |
| Seagrass | 152 | 4/21/16 | 1.1 | 29.87 | 16.2 | 32.2 | 1 | 10.514 | 0.923 |
| Seagrass | 152 | 4/21/16 | 1.1 | 29.87 | 16.2 | 32.2 | 1 | 7.792 | 0.879 |
| Seagrass | 152 | 4/21/16 | 1.1 | 29.87 | 16.2 | 32.2 | 1 | 0.138 | 0.015 |
| Seagrass | 152 | 4/21/16 | 1.1 | 29.87 | 16.2 | 32.2 | 1 | 0.706 | 0.036 |
| Seagrass | 152 | 4/21/16 | 1.1 | 29.87 | 16.2 | 32.2 | 1 | 1.416 | 0.062 |
| Seagrass | 152 | 4/21/16 | 1.1 | 29.87 | 16.2 | 32.2 | 1 | 1.098 | 0.143 |
| Seagrass | 152 | 4/21/16 | 1.1 | 29.87 | 16.2 | 32.2 | 1 | 5.201 | 0.237 |
| Seagrass | 152 | 4/21/16 | 1.1 | 29.87 | 16.2 | 32.2 | 1 | 15.945 | 1.606 |
| Seagrass | 148 | 4/21/16 | 1.9 | 29.87 | 16.2 | 32.2 | 1.4 | 0.551 | 0.030 |
| Seagrass | 148 | 4/21/16 | 1.9 | 29.87 | 16.2 | 32.2 | 1.4 | 0.270 | 0.023 |
| Seagrass | 148 | 4/21/16 | 1.9 | 29.87 | 16.2 | 32.2 | 1.4 | 0.086 | 0.017 |
| Seagrass | 148 | 4/21/16 | 1.9 | 29.87 | 16.2 | 32.2 | 1.4 | 0.000 | 0.001 |
| Bare | 148 | 4/21/16 | 1.9 | 29.87 | 16.2 | 32.2 | 0 | 0.049 | 0.007 |
| Bare | 148 | 4/21/16 | 1.9 | 29.87 | 16.2 | 32.2 | 0 | 3.081 | 0.137 |
| Bare | 148 | 4/21/16 | 1.9 | 29.87 | 16.2 | 32.2 | 0 | 0.507 | 0.056 |
| Bare | 148 | 4/21/16 | 1.9 | 29.87 | 16.2 | 32.2 | 0 | n.d. | n.d. |
| Seagrass | 105 | 6/16/16 | 2.2 | 29.87 | 25.4 | 32.1 | 78 | 2.160 | 0.000 |
| Seagrass | 105 | 6/16/16 | 2.2 | 29.87 | 25.4 | 32.1 | 78 | 1.537 | 0.011 |
| Seagrass | 105 | 6/16/16 | 2.2 | 29.87 | 25.4 | 32.1 | 78 | 0.161 | 0.003 |
| Seagrass | 105 | 6/16/16 | 2.2 | 29.87 | 25.4 | 32.1 | 78 | 6.335 | 0.070 |
| Seagrass | 128 | 6/16/16 | 3.5 | 29.87 | 25.4 | 32.1 | 48 | 1.134 | 0.004 |
| Seagrass | 128 | 6/16/16 | 3.5 | 29.87 | 25.4 | 32.1 | 48 | 0.927 | 0.012 |
| Seagrass | 128 | 6/16/16 | 3.5 | 29.87 | 25.4 | 32.1 | 48 | 0.010 | 0.000 |
| Seagrass | 128 | 6/16/16 | 3.5 | 29.87 | 25.4 | 32.1 | 48 | 0.003 | 0.000 |
| Bare | 105 | 6/16/16 | 2.1 | 29.87 | 25.4 | 32.1 | 0 | 0.005 | 0.000 |
| Bare | 105 | 6/16/16 | 2.1 | 29.87 | 25.4 | 32.1 | 0 | 0.010 | 0.001 |
| Bare | 105 | 6/16/16 | 2.1 | 29.87 | 25.4 | 32.1 | 0 | 0.016 | 0.001 |
| Bare | 105 | 6/16/16 | 2.1 | 29.87 | 25.4 | 32.1 | 0 | 0.010 | 0.000 |
| Bare | 128 | 6/16/16 | 3.3 | 29.87 | 25.4 | 32.1 | 2 | 2.982 | 0.003 |
| Bare | 128 | 6/16/16 | 3.3 | 29.87 | 25.4 | 32.1 | 2 | 0.008 | 0.000 |
| Bare | 128 | 6/16/16 | 3.3 | 29.87 | 25.4 | 32.1 | 2 | 0.006 | 0.000 |
| Bare | 128 | 6/16/16 | 3.3 | 29.87 | 25.4 | 32.1 | 2 | 0.008 | 0.001 |
| Seagrass | 105 | 6/19/16 | 4.3 | 29.8075 | 26.3 | 31.9 | 57 | 40.216 | 0.320 |
| Seagrass | 105 | 6/19/16 | 4.3 | 29.87 | 26.3 | 31.9 | 57 | 24.418 | 0.223 |
| Seagrass | 105 | 6/19/16 | 4.3 | 29.8075 | 26.3 | 31.9 | 57 | 15.502 | 0.109 |
| Seagrass | 105 | 6/19/16 | 4.3 | 29.8075 | 26.3 | 31.9 | 57 | 1.089 | 0.004 |
| Seagrass | 106 | 6/19/16 | 5.0 | 29.8075 | 26.3 | 31.9 | 51 | 1.363 | 0.085 |
| Seagrass | 106 | 6/19/16 | 5.0 | 29.8075 | 26.3 | 31.9 | 51 | 4.823 | 0.041 |
| Seagrass | 106 | 6/19/16 | 5.0 | 29.8075 | 26.3 | 31.9 | 51 | 19.063 | 0.212 |
| Seagrass | 106 | 6/19/16 | 5.0 | 29.87 | 26.3 | 31.9 | 51 | 29.125 | 0.238 |
| Bare | 105 | 6/19/16 | 4.2 | 29.87 | 26.3 | 31.9 | 0 | 0.003 | 0.000 |
| Bare | 105 | 6/19/16 | 4.2 | 29.8075 | 26.3 | 31.9 | 0 | 0.002 | 0.000 |
| Bare | 105 | 6/19/16 | 4.2 | 29.87 | 26.3 | 31.9 | 0 | 0.000 | 0.000 |
| Bare | 105 | 6/19/16 | 4.2 | 29.8075 | 26.3 | 31.9 | 0 | 0.005 | 0.000 |
| Bare | 106 | 6/19/16 | 5.0 | 29.8075 | 26.3 | 31.9 | 0 | 0.101 | 0.002 |
| Bare | 106 | 6/19/16 | 5.0 | 29.8075 | 26.3 | 31.9 | 0 | 0.003 | 0.000 |
| Bare | 106 | 6/19/16 | 5.0 | 29.8075 | 26.3 | 31.9 | 0 | 0.003 | 0.000 |
| Bare | 106 | 6/19/16 | 5.0 | 29.8075 | 26.3 | 31.9 | 0 | 0.002 | 0.000 |
| Seagrass | 106 | 6/20/16 | 3.4 | 29.8075 | 27.8 | n.d. | 28 | 16.439 | 0.141 |
| Seagrass | 106 | 6/20/16 | 3.4 | 29.8075 | 27.8 | n.d. | 28 | 23.327 | 0.190 |
| Seagrass | 106 | 6/20/16 | 3.4 | 29.8075 | 27.8 | n.d. | 28 | 2.877 | 0.030 |
| Seagrass | 106 | 6/20/16 | 3.4 | 30.135 | 27.8 | n.d. | 28 | 0.903 | 0.189 |
| Seagrass | 128 | 6/20/16 | 2.3 | 29.8075 | 27.8 | n.d. | 56 | 98.528 | 0.755 |
| Seagrass | 128 | 6/20/16 | 2.3 | 29.8075 | 27.8 | n.d. | 56 | 40.331 | 0.411 |
| Seagrass | 128 | 6/20/16 | 2.3 | 29.8075 | 27.8 | n.d. | 56 | 44.376 | 1.085 |
| Seagrass | 128 | 6/20/16 | 2.3 | 29.8075 | 27.8 | n.d. | 56 | 5.836 | 0.122 |
| Bare | 106 | 6/20/16 | 3.3 | 29.8075 | 27.8 | n.d. | 0 | 0.004 | 0.001 |
| Bare | 106 | 6/20/16 | 3.3 | 29.8075 | 27.8 | n.d. | 0 | 0.158 | 0.007 |
| Bare | 106 | 6/20/16 | 3.3 | 29.8075 | 27.8 | n.d. | 0 | 0.002 | 0.000 |
| Bare | 106 | 6/20/16 | 3.3 | 29.8075 | 27.8 | n.d. | 0 | 0.004 | 0.000 |
| Bare | 128 | 6/20/16 | 2.2 | 29.8075 | 27.8 | n.d. | 0 | 0.186 | 0.030 |
| Bare | 128 | 6/20/16 | 2.2 | 29.8075 | 27.8 | n.d. | 0 | 1.559 | 0.034 |
| Bare | 128 | 6/20/16 | 2.2 | 29.8075 | 27.8 | n.d. | 0 | 1.217 | 0.034 |
| Bare | 128 | 6/20/16 | 2.2 | 29.87 | 27.8 | n.d. | 0 | 0.542 | 0.027 |
| Seagrass | 105 | 7/14/16 | 2.6 | 30.2025 | 29.1 | 33.5 | n.d. | 0.588 | 0.013 |
| Seagrass | 105 | 7/14/16 | 2.6 | 30.2025 | 29.1 | 33.5 | n.d. | 21.270 | 0.182 |
| Seagrass | 105 | 7/14/16 | 2.6 | 30.2025 | 29.1 | 33.5 | n.d. | 10.022 | 0.107 |
| Seagrass | 105 | 7/14/16 | 2.6 | 30.2025 | 29.1 | 33.5 | n.d. | 4.802 | 0.107 |
| Seagrass | 128 | 7/14/16 | 2.1 | 30.2025 | 29.1 | 33.5 | 47 | 9.305 | 0.132 |
| Seagrass | 128 | 7/14/16 | 2.1 | 30.2025 | 29.1 | 33.5 | 47 | 1.299 | 0.036 |
| Seagrass | 128 | 7/14/16 | 2.1 | 30.2025 | 29.1 | 33.5 | 47 | 0.340 | 0.008 |
| Seagrass | 128 | 7/14/16 | 2.1 | 30.2025 | 29.1 | 33.5 | 47 | 0.007 | 0.000 |
| Bare | 105 | 7/14/16 | 2.7 | 30.2025 | 29.1 | 33.5 | 0 | 0.000 | 0.000 |
| Bare | 105 | 7/14/16 | 2.7 | 30.2025 | 29.1 | 33.5 | 0 | 0.013 | 0.002 |
| Bare | 105 | 7/14/16 | 2.7 | 30.2025 | 29.1 | 33.5 | 0 | 0.000 | 0.000 |
| Bare | 105 | 7/14/16 | 2.7 | 30.2025 | 29.1 | 33.5 | 0 | 0.006 | 0.000 |
| Bare | 128 | 7/14/16 | 2.2 | 30.2025 | 29.1 | 33.5 | 0 | 0.009 | 0.001 |
| Bare | 128 | 7/14/16 | 2.2 | 30.2025 | 29.1 | 33.5 | 0 | 0.008 | 0.000 |
| Bare | 128 | 7/14/16 | 2.2 | 30.2025 | 29.1 | 33.5 | 0 | 0.006 | 0.001 |
| Bare | 128 | 7/14/16 | 2.2 | 30.2025 | 29.1 | 33.5 | 0 | 0.051 | 0.002 |
| Seagrass | 105 | 8/29/16 | 2.2 | 30.2025 | 27.3 | 32 | 71 | 0.797 | 0.000 |
| Seagrass | 105 | 8/29/16 | 2.2 | 30.2025 | 27.3 | 32 | 71 | 0.028 | 0.001 |
| Seagrass | 105 | 8/29/16 | 2.2 | 30.2025 | 27.3 | 32 | 71 | 0.027 | 0.000 |
| Seagrass | 105 | 8/29/16 | 2.2 | 30.2025 | 27.3 | 32 | 71 | 0.033 | 0.000 |
| Seagrass | 128 | 8/29/16 | 2.3 | 30.2025 | 27.3 | 32 | 54 | 0.021 | 0.000 |
| Seagrass | 128 | 8/29/16 | 2.3 | 30.2025 | 27.3 | 32 | 54 | 0.016 | 0.000 |
| Seagrass | 128 | 8/29/16 | 2.3 | 30.2025 | 27.3 | 32 | 54 | 0.071 | 0.001 |
| Seagrass | 128 | 8/29/16 | 2.3 | 30.2025 | 27.3 | 32 | 54 | 0.020 | 0.000 |
| Bare | 105 | 8/29/16 | 2.2 | 30.2025 | 27.3 | 32 | 0 | 0.014 | 0.000 |
| Bare | 105 | 8/29/16 | 2.2 | 30.2025 | 27.3 | 32 | 0 | 0.172 | 0.000 |
| Bare | 105 | 8/29/16 | 2.2 | 30.2025 | 27.3 | 32 | 0 | 0.000 | 0.000 |
| Bare | 105 | 8/29/16 | 2.2 | 30.2025 | 27.3 | 32 | 0 | 0.017 | 0.000 |
| Bare | 128 | 8/29/16 | 2.3 | 30.2025 | 27.3 | 32 | 0 | 0.049 | 0.001 |
| Bare | 128 | 8/29/16 | 2.3 | 30.2025 | 27.3 | 32 | 0 | 0.012 | 0.001 |
| Bare | 128 | 8/29/16 | 2.3 | 30.2025 | 27.3 | 32 | 0 | 0.016 | 0.000 |
| Bare | 128 | 8/29/16 | 2.3 | 30.2025 | 27.3 | 32 | 0 | 0.019 | 0.000 |
| Seagrass | 128 | 8/30/16 | 2.0 | 29.9525 | 28.5 | 33.7 | 59 | 1.456 | 0.052 |
| Seagrass | 128 | 8/30/16 | 2.0 | 29.9525 | 28.5 | 33.7 | 59 | 0.040 | 0.005 |
| Seagrass | 128 | 8/30/16 | 2.0 | 29.9525 | 28.5 | 33.7 | 59 | 2.323 | 0.112 |
| Seagrass | 128 | 8/30/16 | 2.0 | 29.9525 | 28.5 | 33.7 | 59 | 0.047 | 0.003 |
| Seagrass | 148 | 8/30/16 | 1.8 | 30.2025 | 28.5 | 33.7 | 56 | 0.074 | 0.003 |
| Seagrass | 148 | 8/30/16 | 1.8 | 29.9525 | 28.5 | 33.7 | 56 | 0.050 | 0.008 |
| Seagrass | 148 | 8/30/16 | 1.8 | 29.9525 | 28.5 | 33.7 | 56 | 0.057 | 0.007 |
| Seagrass | 148 | 8/30/16 | 1.8 | 29.9525 | 28.5 | 33.7 | 56 | 0.050 | 0.008 |
| Bare | 128 | 8/30/16 | 2.1 | 29.9525 | 28.5 | 33.7 | 0 | 0.067 | 0.003 |
| Bare | 128 | 8/30/16 | 2.1 | 29.9525 | 28.5 | 33.7 | 0 | 0.067 | 0.002 |
| Bare | 128 | 8/30/16 | 2.1 | 30.2025 | 28.5 | 33.7 | 0 | 0.052 | 0.004 |
| Bare | 128 | 8/30/16 | 2.1 | 29.9525 | 28.5 | 33.7 | 0 | 0.112 | 0.005 |
| Bare | 148 | 8/30/16 | 1.7 | 29.9525 | 28.5 | 33.7 | 0 | 0.000 | 0.000 |
| Bare | 148 | 8/30/16 | 1.7 | 29.9525 | 28.5 | 33.7 | 0 | 0.065 | 0.004 |
| Bare | 148 | 8/30/16 | 1.7 | 29.9525 | 28.5 | 33.7 | 0 | 0.705 | 0.070 |
| Bare | 148 | 8/30/16 | 1.7 | 29.9525 | 28.5 | 33.7 | 0 | 0.058 | 0.002 |
| Seagrass | 105 | 10/25/16 | 2.7 | 29.9525 | 15.1 | 30.6 | 11 | 0.005 | 0.000 |
| Seagrass | 105 | 10/25/16 | 2.7 | 29.9525 | 15.1 | 30.6 | 11 | 0.055 | 0.016 |
| Seagrass | 105 | 10/25/16 | 2.7 | 29.9525 | 15.1 | 30.6 | 11 | 0.944 | 0.094 |
| Seagrass | 105 | 10/25/16 | 2.7 | 29.9525 | 15.1 | 30.6 | 11 | 0.539 | 0.046 |
| Seagrass | 106 | 10/25/16 | 2.2 | 29.9525 | 15.1 | 30.6 | 37 | 0.169 | 0.017 |
| Seagrass | 106 | 10/25/16 | 2.2 | 29.9525 | 15.1 | 30.6 | 37 | 0.649 | 0.028 |
| Seagrass | 106 | 10/25/16 | 2.2 | 29.9525 | 15.1 | 30.6 | 37 | 1.044 | 0.000 |
| Seagrass | 106 | 10/25/16 | 2.2 | 29.9525 | 15.1 | 30.6 | 37 | 0.165 | 0.017 |
| Bare | 105 | 10/25/16 | 2.8 | 29.9525 | 15.1 | 30.6 | 0 | 0.012 | 0.000 |
| Bare | 105 | 10/25/16 | 2.8 | 29.9525 | 15.1 | 30.6 | 0 | 0.679 | 0.070 |
| Bare | 105 | 10/25/16 | 2.8 | 29.9525 | 15.1 | 30.6 | 0 | 1.791 | 0.182 |
| Bare | 105 | 10/25/16 | 2.8 | 29.9525 | 15.1 | 30.6 | 0 | 0.017 | 0.001 |
| Bare | 106 | 10/25/16 | 2.3 | 29.9525 | 15.1 | 30.6 | 0 | 0.006 | 0.001 |
| Bare | 106 | 10/25/16 | 2.3 | 29.9525 | 15.1 | 30.6 | 0 | 0.084 | 0.009 |
| Bare | 106 | 10/25/16 | 2.3 | 29.9525 | 15.1 | 30.6 | 0 | 0.000 | 0.002 |
| Bare | 106 | 10/25/16 | 2.3 | 29.9525 | 15.1 | 30.6 | 0 | 0.137 | 0.021 |
| Seagrass | 105 | 10/27/16 | 3.7 | 29.9525 | 16.9 | 31.3 | 34 | 0.771 | 0.104 |
| Seagrass | 105 | 10/27/16 | 3.7 | 29.9525 | 16.9 | 31.3 | 34 | 0.000 | 0.006 |
| Seagrass | 105 | 10/27/16 | 3.7 | 29.9525 | 16.9 | 31.3 | 34 | 0.000 | 0.000 |
| Seagrass | 105 | 10/27/16 | 3.7 | 29.9525 | 16.9 | 31.3 | 34 | 0.059 | 0.024 |
| Seagrass | 128 | 10/27/16 | 3.0 | 29.9525 | 16.9 | 31.3 | 4 | 0.818 | 0.081 |
| Seagrass | 128 | 10/27/16 | 3.0 | 29.9525 | 16.9 | 31.3 | 4 | 0.000 | 0.001 |
| Seagrass | 128 | 10/27/16 | 3.0 | 29.9525 | 16.9 | 31.3 | 4 | n.d | n.d. |
| Seagrass | 128 | 10/27/16 | 3.0 | 29.9525 | 16.9 | 31.3 | 4 | n.d. | n.d. |
| Bare | 105 | 10/27/16 | 3.8 | 29.9525 | 16.9 | 31.3 | 0 | 2.048 | 0.228 |
| Bare | 105 | 10/27/16 | 3.8 | 29.9525 | 16.9 | 31.3 | 0 | 0.048 | 0.013 |
| Bare | 105 | 10/27/16 | 3.8 | 29.9525 | 16.9 | 31.3 | 0 | 0.000 | 0.004 |
| Bare | 105 | 10/27/16 | 3.8 | 29.9525 | 16.9 | 31.3 | 0 | 0.179 | 0.000 |
| Bare | 128 | 10/27/16 | 3.0 | 29.9525 | 16.9 | 31.3 | 0 | 0.018 | 0.012 |
| Bare | 128 | 10/27/16 | 3.0 | 29.9525 | 16.9 | 31.3 | 0 | 0.000 | 0.001 |
| Bare | 128 | 10/27/16 | 3.0 | 29.9525 | 16.9 | 31.3 | 0 | 0.000 | 0.031 |
| Bare | 128 | 10/27/16 | 3.0 | 29.9525 | 16.9 | 31.3 | 0 | 0.052 | 0.026 |

^A^ Site locations available from McGlathery^9^

**Selecting Meadow Maps Generated Using Kriging:**

**Table S5. Root mean square error cross-validation to identify optimal stock change distribution maps (Figure 3 in the main text); Note that the uppermost 2 cm of the bed in 2016 was shown as a separate layer to represent apparent bed accretion between 2013-2016, but isolating the seagrass-enhanced C_org_ fraction does not require quantifying bed accretion over time (see Figure 1 in the main text); ABG = aboveground biomass; Exp. = Exponential.**

| Model | Direction | 2013 net increase | 2016 net increase | 2016  accretion | AGB |
| --- | --- | --- | --- | --- | --- |
| Stable | Isotropic | 1.47E-03 | 1.76E-03 | 1.95E-03 | 1.41E-03 |
|  | Anisotropic | 1.55E-03 | 1.73E-03 | 1.91E-03 | 1.43E-03 |
| Circular | Isotropic | 1.43E-03 | 1.76E-03 | 2.00E-03 | 1.41E-03 |
|  | Anisotropic | 1.50E-03 | 1.74E-03 | 2.01E-03 | 1.39E-03 |
| Spherical | Isotropic | 1.44E-03 | 1.76E-03 | 1.99E-03 | 1.41E-03 |
|  | Anisotropic | 1.54E-03 | 1.73E-03 | 2.01E-03 | 1.39E-03 |
| Exp. | Isotropic | 1.45E-03 | 1.76E-03 | 1.92E-03 | 1.42E-03 |
|  | Anisotropic | 1.41E-03 | 1.73E-03 | 1.91E-03 | 1.35E-03 |
| Gaussian | Isotropic | 1.45E-03 | 1.76E-03 | 1.99E-03 | 1.41E-03 |
|  | Anisotropic | 1.55E-03 | 1.73E-03 | 1.99E-03 | 1.43E-03 |


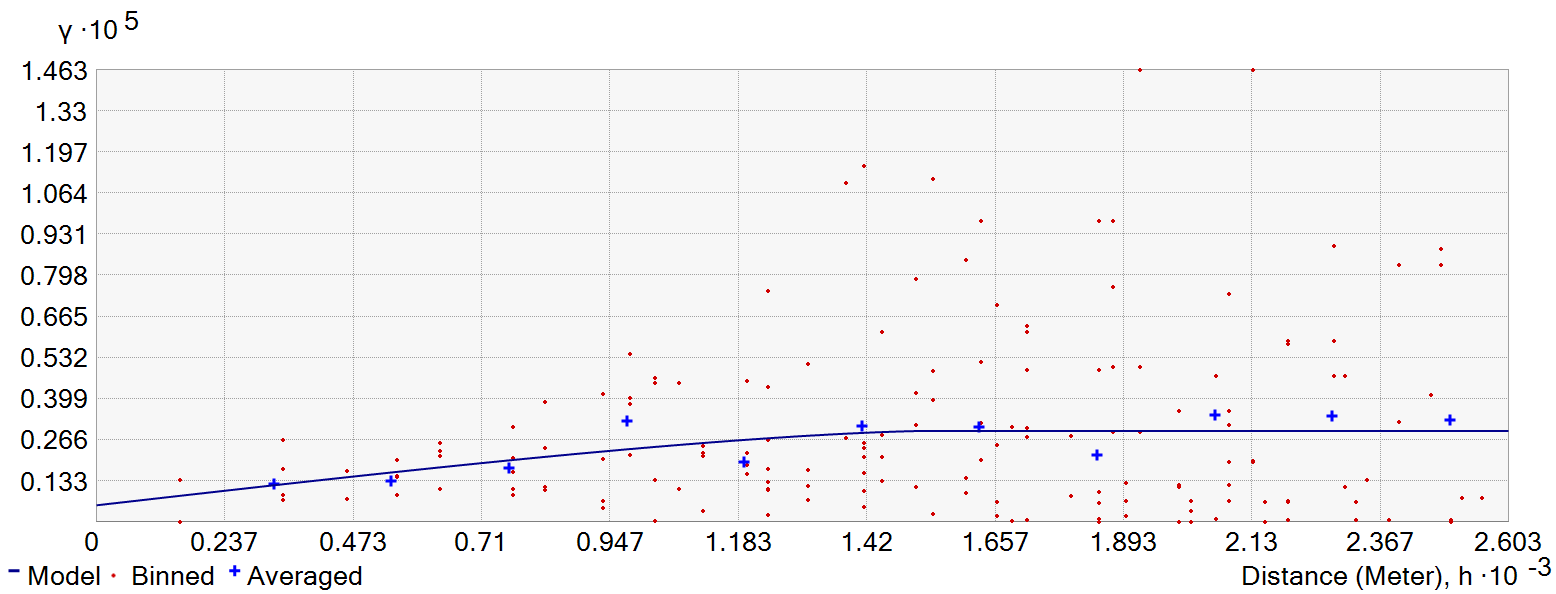


**Figure S1. 12-cm net sediment C_org_ accumulation in 2013 (Ordinary kriging, Circular model, Isotropic); Nugget = 5.29e-7; Major range = 1535.7; Partial sill = 2.41e-6.**


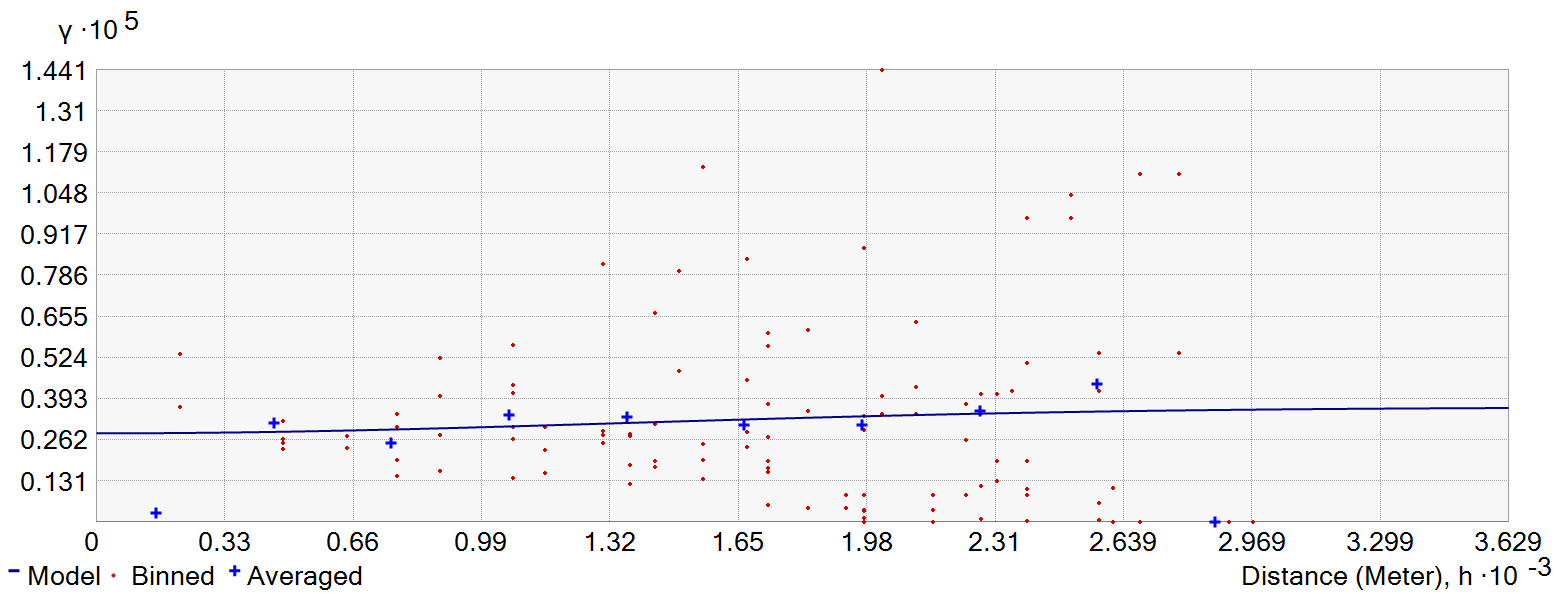


**Figure S2. 12-cm net sediment C_org_ accumulation in 2016 (Ordinary kriging, Gaussian model, Anisotropic); Nugget = 2.814e-6; Major range = 3310.3; Partial sill = 8.23e-7.**


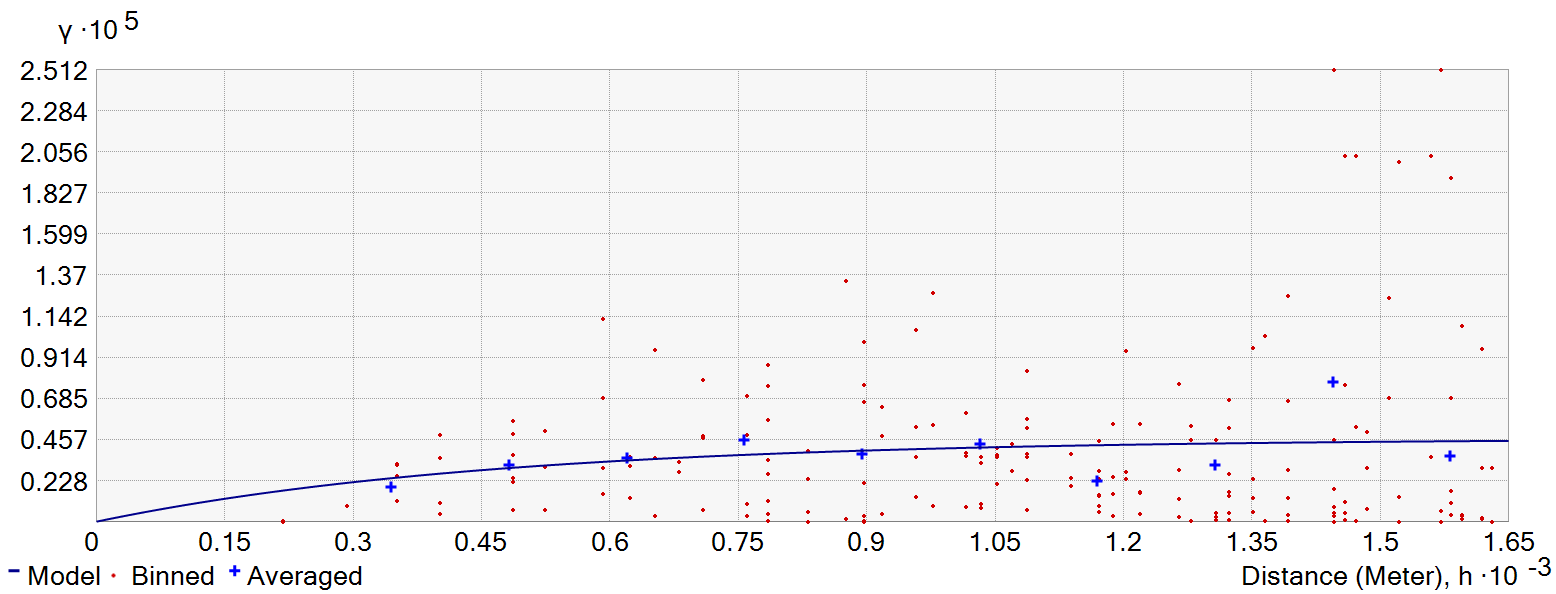


**Figure S3. Net sediment C_org_ accumulation 2-cm 2016 accreted interval (Ordinary kriging, Exponential model, Isotropic); Nugget = 0; Major range = 1399.4; Partial sill = 4.62e-6.**

**Supplement References:**

1. Emmer, I., et al. Methodology for Tidal Wetland and Seagrass Restoration. Verified Carbon Standard, VM0033 Version 1.0. https://verra.org/methodology/vm0033-methodology-for-tidal-wetland-and-seagrass-restoration-v1-0/ (2015). [↑](#endnote-ref-1)
2. Fourqurean, J. W., et al. Seagrass ecosystems as a globally significant carbon stock. *Nat. Geosci.* **5**, 505-509 (2012). [↑](#endnote-ref-2)
3. Johannessen, S. C., & Macdonald, R. W. Geoengineering with seagrasses: is credit due

   where credit is given? *Env. Res. Letters* **11**, 113001; 10.1088/1748-9326/11/11/113001

   (2016). [↑](#endnote-ref-3)
4. Saderne, V., et al. Role of carbonate burial in “blue carbon” budgets. *Nat. Commun.* **10**, 1106; [10.1038/s41467-019-08842-6](https://doi.org/10.1038/s41467-019-08842-6) (2019). [↑](#endnote-ref-4)
5. Lefebvre, A., Thompson, C. E. L., & Amos, C. L. Influence of *Zostera marina* canopies on unidirectional flow, hydraulic roughness and sediment movement. *Continental Shelf Res.* **30**, 1783-1794 (2010). [↑](#endnote-ref-5)
6. Oreska, M. P. J., et al. Comment on Geoengineering with seagrasses: is credit due where credit is given? *Environ. Res. Lett.* **13**(3), 038001; 10.1088/1748-9326/aaae72 (2018). [↑](#endnote-ref-6)
7. Greiner, J. T., McGlathery, K. J., Gunnell, J., & McKee, B. A. Seagrass restoration enhances “blue carbon” sequestration in coastal waters. *PLoS ONE* **8**(8), e72469; DOI:[10.1371/journal.pone.0072469](http://dx.doi.org/10.1371/journal.pone.0072469) (2013). [↑](#endnote-ref-7)
8. Oreska, M. P. J., Wilkinson, G. M., McGlathery, K. J., Bost, M., & McKee, B. A. Non-seagrass carbon contributions to seagrass sediment blue carbon. *Limnol. Oceanogr.* **63**(S1), S3-S18 (2018). [↑](#endnote-ref-8)
9. McGlathery, K. J. GPS locations of seagrass sites in Hog Island Bay and South Bay, VA 2010-2017. Environmental Data Initiative. <http://doi.org/10.6073/pasta/300eb256c8e219c81e5cf426a3098fdf> (2017). [↑](#endnote-ref-9)
10. Oreska, M. P. J., McGlathery, K. J., & Porter, J. H. Seagrass blue carbon accumulation at the meadow-scale. *PLoS ONE* **12**(4), e0176630; 10.1371/journal.pone.0176630 (2017). [↑](#endnote-ref-10)
11. Thomas, E. Influence of *Zostera marina* on wave dynamics, sediment suspension, and bottom boundary layer development within a shallow coastal bay. Thesis submitted to the University of Virginia 28-30 (2014). [↑](#endnote-ref-11)
12. McGlathery, K. J. Above- and Below-Ground Biomass and Canopy Height of Seagrass in Hog Island Bay and South Bay, VA 2007-2017. Environmental Data Initiative.

    <http://dx.doi.org/10.6073/pasta/09a0ce35bb3fc72113b5a16ad5b0d6bd> (2017). [↑](#endnote-ref-12)
13. McGlathery K. J*.* Carbon and Nitrogen in Seagrass Tissue from Virginia Coastal Bays, 2010-2017. Environmental Data Initiative. <http://dx.doi.org/10.6073/pasta/b4d1f74041d329386591a32e9ea202b2> (2017). [↑](#endnote-ref-13)
14. McGlathery, K. J. Density of seagrass in Hog Island Bay and South Bay, VA 2007-2017. Environmental Data Initiative. <http://doi.org/doi:10.6073/pasta/5a6ea442cf59cabb3112bb634a968ae5> (2017). [↑](#endnote-ref-14)
